# Supplementary material for: Smart phone-based transcutaneous electrical acupoint stimulation as adjunctive therapy for hypertension (STAT-H trial): protocol for a cluster randomised controlled trial
Source: BMJ Open. 2022 Jul 28;12(7):e058172. doi: 10.1136/bmjopen-2021-058172 (PMC9341214; doi:10.1136/bmjopen-2021-058172)
Supplement: Supplementary data [file bmjopen-2021-058172supp001.pdf]

eTable 1 The list of Community Health Service Centers

**Number**	**Institution**	**District**
1	Jianguomen Community Health Service Center	Dongcheng District
2	Hepingli Community Health Service Center	Dongcheng District
3	Shichahai Community Health Service Center	Xicheng District
4	Yuetan Community Health Service Center	Xicheng District
5	Sunhe Community Health Service Center	Chaoyang District
6	Cuigezhuang Second Community Health Service Center	Chaoyang District
7	Nanyuan Community Health Service Center	Fengtai District
8	Dahongmen Community Health Service Center	Fengtai District
9	Jindingjie Community Health Service Center	Shijingshan District
10	Guangning Street Community Health Service Center	Shijingshan District
11	Qinglongqiao Community Health Service Center	Haidian District
12	Malanwa Community Health Service Station	Haidian District
13	Tianzhu Community Health Service Center	Shunyi District
14	Zhaoquanying Community Health Service Center	Shunyi District
15	Yongshun Community Health Service Center	Tongzhou District
16	Taihu Community Health Service Center	Tongzhou District
17	Xihongmen Community Health Service Center	Daxing District
18	Changziying Community Health Service Center	Daxing District
19	Liangxiang Community Health Service Station	Fangshan District
20	Changyang Community Health Service Center	Fangshan District

21	Junzhuang Community Health Service Center	Mentougou District
22	Zaitang Community Health Service Center	Mentougou District
23	Shahe Community Health Service Center	Changping District
24	Huilongguan Community Health Service Center	Changping District
25	Huangsongyu Community Health Service Center	Pinggu District
26	Yukou Community Health Service Center	Pinggu District
27	Henanzhai Community Health Service Center	Miyun District
28	Mujianyu Community Health Service Center	Miyun District
29	Yangsong Community Health Service Center	Huairou District
30	Miaocheng Community Health Service Center	Huairou District
31	Zhangshanying Community Health Service Center	Yanqing District
32	Yongning Community Health Service Center	Yanqing District
