# Supplementary material for: Smart phone-based transcutaneous electrical acupoint stimulation as adjunctive therapy for hypertension (STAT-H trial): protocol for a cluster randomised controlled trial
Source: BMJ Open. 2022 Jul 28;12(7):e058172. doi: 10.1136/bmjopen-2021-058172 (PMC9341214; doi:10.1136/bmjopen-2021-058172)
Supplement: Author's manuscript [file bmjopen-2021-058172.draft_revisions.pdf]

BMJ Open is committed to open peer review. As part of this commitment we make the peer review history of every article we publish publicly available.

When an article is published we post the peer reviewers' comments and the authors' responses online. We also post the versions of the paper that were used during peer review. These are the versions that the peer review comments apply to.

The versions of the paper that follow are the versions that were submitted during the peer review process. They are not the versions of record or the final published versions. They should not be cited or distributed as the published version of this manuscript.

BMJ Open is an open access journal and the full, final, typeset and author-corrected version of record of the manuscript is available on our site with no access controls, subscription charges or pay-per-view fees (http://bmjopen.bmj.com).

If you have any questions on BMJ Open's open peer review process please email info.bmjopen@bmj.com

BMJ Open

Smart phone-based Transcutaneous electrical acupoint stimulation as Adjunctive Therapy for Hypertension (STAT-H trial): protocol for a cluster randomised controlled trial

Journal:	*BMJ Open*
Manuscript ID	bmjopen-2021-058172
Article Type:	Protocol
Date Submitted by the Author:	10-Oct-2021
Complete List of Authors:	Tu, Jian Feng; Beijing University of Chinese Medicine, School of Acupuncture-Moxibustion and Tuina<br>Kang, Si-Bo; Beijing University of Chinese Medicine<br>Wang, Li-Qiong; Beijing University of Chinese Medicine<br>YAN, Shiyan; Beijing University of Chinese Medicine<br>Yan, Chao-qun; Dongzhimen Hospital, Beijing University of Chinese Medicine<br>Su, Xin-Tong; Beijing University of Chinese Medicine<br>Shi, Guangxia; Beijing University of Chinese Medicine<br>Mi, Bao-Hong; Beijing University of Chinese Medicine<br>Lin, Ying; Beijing University of Chinese Medicine<br>Wang, Yu; Beijing University of Chinese Medicine, Acupuncture and Moxibustion Department<br>Li, He-Wen; Beijing University of Chinese Medicine<br>Wang, Xue-Zhou; Beijing University of Chinese Medicine<br>Wang, Xiao; Beijing University of Chinese Medicine<br>Yang, Jing-Wen; Beijing University of Chinese Medicine<br>Liu, Cun-Zhi; Beijing University of Chinese Medicine, Department of Acupuncture and Moxibustion
Keywords:	COMPLEMENTARY MEDICINE, Hypertension < CARDIOLOGY, Clinical trials < THERAPEUTICS

SCHOLARONE™
Manuscripts

Smart phone-based Transcutaneous electrical acupoint stimulation as Adjunctive Therapy for Hypertension (STAT-H trial): protocol for a cluster randomised controlled trial

Jian-Feng Tu[1, #], Si-Bo Kang[1, #], Li-Qiong Wang[1], Shi-Yan Yan[1], Chao-Qun Yan[2], Xin-Tong Su[1], Guang-Xia Shi[1], Bao-Hong Mi[1], Ying Lin[1], Yu Wang[1], He-Wen Li[1], Xue-Zhou Wang[1], Xiao Wang[1], Jing-Wen Yang[1], Cun-Zhi Liu[1, *]

1 International Acupuncture and Moxibustion Innovation Institute, School of Acupuncture-Moxibustion and Tuina, Beijing University of Chinese Medicine, Beijing, China

2 Department of Acupuncture and Moxibustion, Dongzhimen Hospital, Beijing University of Chinese Medicine, Beijing, China

[#] The two authors contributed equally.

*Corresponding author: Cun-Zhi Liu

International Acupuncture and Moxibustion Innovation Institute, School of Acupuncture-Moxibustion and Tuina, Beijing University of Chinese Medicine, No. 11, Bei San Huan Dong Lu, Chaoyang District, Beijing 100029, China.

E-mail address: lcz_tg@126.com

Running title: Smart phone-based TEAS as Adjunctive Therapy for Hypertension

Disclaimers: The authors have no conflicts of interest to declare.

For peer review only - http://bmjopen.bmj.com/site/about/guidelines.xhtml

Word count: 2905 words

Number of figures: 3

Number of tables: 1

ORCID

Cun-Zhi Liu https://orcid.org/0000-0001-8031-5667

Jian-Feng Tu http://orcid.org/0000-0003-2887-3383

For peer review only

For peer review only - http://bmjopen.bmj.com/site/about/guidelines.xhtml

ABSTRACT

Introduction: Hypertension is a common risk factor for cardiovascular disease. Transcutaneous electrical acupoint stimulation (TEAS) may be effective for hypertension, but the evidence remains limited. The aim of this study is to evaluate the effectiveness and safety of the smart phone-based TEAS as adjunctive therapy for hypertension.

Methods and analysis: This study is a 52-week cluster randomised controlled trial with 1600 hypertension patients in 32 community health service centers. Patients who meet the inclusion criteria will be randomised into usual care group or TEAS group in a 1:1 ratio. All patients will be provided with usual care as recommended by the guidelines. In addition to this, patients in the TEAS group will receive non-invasive acupoint electrical stimulation for 30 minutes at home, 4 times weekly for 12 weeks. The primary outcome will be the mean difference in the changes in office systolic blood pressure from baseline to 12 weeks between TEAS and usual care groups. Secondary outcomes will include the change of mean diastolic blood pressure, proportion of patients with controlled blood pressure (blood pressure ＜ 140/90 mmHg), proportion of patients taking antihypertensive drugs, change in number of antihypertensive drugs, and changes in 12-Item Short-Form. Tertiary outcomes will include change in body mass index, change in waist circumference, physical activity, and medication adherence. Safety outcomes will be any serious adverse events and clinical events.

Ethics and dissemination: This study has been approved by ethics committee of

For peer review only - http://bmjopen.bmj.com/site/about/guidelines.xhtml

Beijing University of Chinese Medicine (No. 2020BZHYLL0104). Written informed consent will be obtained from all patients before randomisation. Trial results will be disseminated in peer-reviewed publications.

Trial registration: ChiCTR2000039400.

Keywords: Transcutaneous electrical acupoint stimulation; hypertension; usual care; cluster randomised controlled trial; protocol

For peer review only

Strengths and limitations of this study

- This is the first randomised trial to investigate the effectiveness and safety of smart phone-based TEAS as adjunctive therapy for hypertension with adequate power.
- This community-based cluster trial could lower the risk of contamination as much as possible and overcome the traditionally low detection of hypertension in hospital-based studies.
- The smart phone-based TEAS can be received through at home by the patients themselves to reduce the burden of logistics and lost wages.
- The background of TEAS software would recognise patients with poor compliance real-timely and remind them of receiving TEAS to improve the adherence rate.
- Surrogate outcome, not clinical event, is used as primary outcome and blinding for patients was not feasible in this study.

For peer review only - http://bmjopen.bmj.com/site/about/guidelines.xhtml

INTRODUCTION

Cardiovascular disease is the most common cause of premature death and disability worldwide, and hypertension is the most common risk factor for cardiovascular disease.[1] World Health Organization (WHO) suggested that the global prevalence of hypertension was 20% for females and 25% for males.[2] Moreover, according to the latest American College of Cardiology/American Heart Association (ACC/AHA) guideline,[3] the prevalence of hypertension increased to nearly 50%.[4-6] Hypertension accounted for 9.4 million deaths and 7.0% of global disability-adjusted life-years (DALYs).[7] The total costs of hypertension will increase to an estimated $274 billion by 2030.[8]

Lifestyle modifications are recommended by all guidelines in different countries,[3 9 10] while most patients are unable to sustain lifestyle changes. Antihypertensive drugs are widely prescribed for patients with hypertension. However, fewer than 20% of patients with hypertension have their blood pressure controlled.[10] The reasons for low control rate of blood pressure include the poor adherence of medicine and the burden of logistics. Identifying non-pharmaceutical therapy with well adherence that can be done at home is urgent needed to reduce blood pressure in patients with hypertension.

Acupuncture, as a non-pharmaceutical therapy, was used for several cardiovascular diseases.[11 12] Previous studies indicated that acupuncture could decrease blood pressure[13 14] and a recent systematic review found that acupuncture as adjunctive

For peer review only - http://bmjopen.bmj.com/site/about/guidelines.xhtml

therapy may be effective for hypertension.[15] However, acupuncture also has the same limitation of logistical burdens as the antihypertensive drugs. Transcutaneous electrical acupoint stimulation (TEAS), a non-invasive acupuncture therapy, has similar effect with acupuncture and can be performed at home by the patients themselves. Our pilot study found that TEAS added to usual care for patients with hypertension was feasible and may be a potential treatment option.[16] The aim of this cluster randomised controlled trial is to evaluate the effectiveness and safety of the smart phone-based TEAS as adjunctive therapy for hypertension.

METHODS

Study Design

This two-arm, open-label, cluster randomised controlled trial will be conducted at 32 community health service centers in China. The total trial period was 52 weeks, including 12 weeks of treatment and 40 weeks of follow-up. The study protocol (version number: 2.0; June 13 2020) has been registered on Chinese clinical trial registry (No. ChiCTR2000039400). The protocol will be reported following SPIRIT guideline.[17] Figure 1 shows the flow diagram of the trial.

Study recruitment

There are 16 districts and 346 community health service centers in Beijing, China. Of them, 32 community health service centers (Supplement 1), 2 centers each district, will be recruited through *Beijing Administration of Traditional Chinese Medicine* and

For peer review only - http://bmjopen.bmj.com/site/about/guidelines.xhtml

Beijing Association of Acupuncture-Moxibustion. Patients will be diagnosed as hypertension according to the European guideline[9] and Chinese guideline[10]. About 50 patients will be enrolled in each center by their family doctors. The family doctors will conduct preliminary screening to exclude ineligible patients, then the office blood pressure will be measured in the consulting room by clinical research coordinators to determine whether the patients will be finally included or not.

Participants

Inclusion criteria:

1. Office systolic blood pressure (SBP) from 140 to 159 mm Hg and/or diastolic blood pressure (DBP) from 90 to 99 mm Hg for patients who did not take antihypertensive drugs previously, or Office SBP from 120 to 159 mm Hg and/or DBP from 80 to 99 mm Hg for patients who do not changing antihypertensive drugs at least 1 month
2. Aged between 18 and 65 years (male or female)
3. Answering and completing the questionnaires
4. Using smart phone
5. Written informed consent

Exclusion criteria:

1. Other diseases that can affect the blood pressure, including but not limited to diseases of renal parenchyma, renal artery stenosis, obstructive sleep apnea syndrome, and primary hyperaldosteronism

For peer review only - http://bmjopen.bmj.com/site/about/guidelines.xhtml

2. Receiving medicines that affect blood pressure except antihypertensive drugs in last month, including but not limited to glucocorticoids and central nervous system inhibitors

3. Contraindications for the use of acupoint stimulators: wearing a pacemaker and other implanted medical devices; epilepsy; scars, bruises, scratches or inflammation on the skin of the acupoints; allergic to electrodes

4. Uncontrolled diabetes (HbA1c ⩾ 6. 5%)

5. Drug or alcohol abuse

6. Pregnant or breastfeeding

7. Participated in other clinical trials in the past 3 months

Randomization and masking

The unit of randomization will be the community health service centers. Thirty-two centers will be randomised to either TEAS or usual care group in a 1:1 ratio with a central randomization system. All patients within the defined center will receive the same allocated treatment. Each center will not be randomised until total 50 patients are recruited to avoid selection bias. The randomization sequence will be generated by an independent statistician with the Stata 12.0 software and will be stratified by districts. The patients and the acupuncturists who teach patients how to locate acupoints and to receive TEAS at home will not be blinded. However, the family doctors who prescribe antihypertensive drugs, outcome assessors and statisticians will be blinded.

For peer review only - http://bmjopen.bmj.com/site/about/guidelines.xhtml

Interventions

Usual care group

All patients will be provided usual care at the respective community health service center, in conjunction with any ongoing care that they are receiving from their family doctor. Patients at high or very high cardiovascular risk will receive antihypertensive drugs according to the recommendation of the guidelines,[9 10] including diuretics, β-blockers, calcium channel blockers, angiotensin receptor blockers, and angiotensin-converting enzyme inhibitors. The choice of antihypertensive drugs will be individualised, with consideration of patients' coexisting conditions and adverse effects to the medications. Research team will engage in consistent communication with each patient's family doctor, including a standard letter conveying the reminder that the doctor is free to implement any additional therapies that were deemed to be appropriate, including increase or decrease the type and dosage of antihypertensive drugs. Research team will also send education materials to all patients. Content will include information about hypertension, its clinical features, epidemiology, diagnostic criteria, and treatment options, as well as the role and suggestions of lifestyle intervention in hypertension.

TEAS group

In addition to the usual care, the patients in the TEAS group will receive 48 sessions of TEAS treatment (4 times weekly for 12 weeks). TEAS will be performed using a

For peer review only - http://bmjopen.bmj.com/site/about/guidelines.xhtml

portable instrument (SDP-310, Hwato, Suzhou Medical Appliances Co, Ltd., Suzhou, China) with a pair of rounded electrodes (diameter = 38 mm) through smart phone. The adhesive electrodes will be applied to the skin as illustrated in Figure 2 after the skin sites are cleaned with alcohol to avoid any barrier conduction of the electrical current. The instrument has ten levels of stimulus intensities, and patients asked to increase the stimulus intensity until local, rhythmic contractions of the muscle are obtained without producing pain. According to the previous studies,[16 18-20] bilateral *Hegu* (LI4), *Quchi* (LI11), *Zusanli* (ST36), and *Taichong* (LR3) will be used in our trial. At the first day, the electrodes will be placed at the ipsilateral LI4 and LI11, and the appropriate stimulus intensity will last for 15 minutes. Then the opposite arm will be stimulated in the same way for other 15 minutes. On the third day, the ST36 and LR3 will be stimulated for 15 minutes per leg in the same way. Two pairs of acupoints (LI4-LI11 and ST36-LR3) will be stimulated alternatively every other day. Locations of acupoints are shown in Table 1. The acupuncturists will train the patients on how to locate the acupoints and use the instrument face-to-face. Paper instructions and acupoint maps will also be provided to help patients to locate the acupoints at home. The date, duration, and specific acupoints of each acupoint stimuli will be recorded real-timely by TEAS software. Thus, the investigator could recognise patients with poor compliance in a timely manner and prompt them to complete the predefined 48 sessions.

Outcomes

For peer review only - http://bmjopen.bmj.com/site/about/guidelines.xhtml

The primary outcome will be the mean difference in the changes in office SBP from baseline to 12 weeks between TEAS and usual care groups. SBP will be measured as per the WHO STEPS protocol to ensure consistency of recordings, and an automated monitor (HEM-7136, Omron Corporation, Kyoto, Japan) will be used at each visit. Patients will be asked to rest quietly for at least 5 minutes in the sitting position before measuring blood pressure. Blood pressure of both arms will be measured at baseline with the upper arm at heart level, and the arm with higher blood pressure will be the one measured throughout the entire study. At each time point, the blood pressure measurement will be repeated 3 times every 5 minutes, and the last two will be averaged.[21 22]

Secondary outcomes will include differences in the changes in office DBP between the TEAS and usual care groups; proportion of patients with well controlled blood pressure (office blood pressure $<$ 140/90 mmHg); proportion of patients taking antihypertensive drugs; differences in the changes in number of antihypertensive drugs; and differences in the changes in 12-Item Short-Form (SF-12).[23] Tertiary outcomes will include change in body mass index (BMI); change in waist circumference; physical activity assessed using International Physical Activity Questionnaire (IPAQ);[24] medication adherence assessed using the 8-item Morisky Medication Adherence Scale 8 (MMAS-8)[25] in patients taking antihypertensive drugs in both groups; and TEAS adherence assessed using number of sessions completed in TEAS group. All secondary outcomes and tertiary outcomes will be measured at

For peer review only - http://bmjopen.bmj.com/site/about/guidelines.xhtml

weeks 12, 26 and 52. Moreover, blinding test of outcome assessors will be measured at week 12.

Safety outcomes will be any serious adverse events and clinical events (death, myocardial infarction, stroke, and cardiovascular-related hospitalisations) at weeks 12, 26 and 52 in both groups. The schedule of enrolment, intervention and assessments is shown in Figure 3.

Sample size calculation

The effective sample size (ESS) of 392 patients in each group is calculated for SBP on the basis of an alpha level of 0.05 (two-sided), a beta level of 0.80, an expected between-group difference of 6 mm Hg,[16] a standard deviation of 10 mm Hg, and a superiority margin of 4 mm Hg,[26] if randomization takes place at the individual level. With average 40 patients per community and an intra-cluster correlation (ICC) of 0.01,[27] the ESS should be multiplied by a design effect of 1.39, leading to 560 patients in 14 communities per group (1120 patients in 28 communities in total). As our experience suggests that up to 10% of the randomised communities and up to 20% of the recruited patients may drop out of the study, we aim to recruit 32 communities (16 per group) including 50 patients each, resulting in a target sample size of 1600 recruited patients in total (800 per group).

Statistical analysis

For peer review only - http://bmjopen.bmj.com/site/about/guidelines.xhtml

A full analysis set, a per-protocol set, and a safety set will be used in this trial. The full analysis set will consist of all randomised patients according to the intention-to-treat principle. The full analysis set will be the primary analysis, and all analyses will be conducted for this population if not otherwise stated. The per-protocol set will include those who complete all follow-ups on time and without major violations. Major violations include but are not limited to not ineligibility according inclusion and exclusion criteria, receiving medicines that affect blood pressure except antihypertensive drugs, or not completing ⩽ 38 sessions TEAS (80% of prespecified 48 sessions). The per-protocol set will be the secondary analysis set and will be used for the sensitivity analyses for primary outcome. All those who receive at least one session of TEAS will be defined as the safety set, which is used for the safety analyses.

Baseline characteristics will be summarised by groups. Continuous variables will be described using the mean (standard deviation), or the median (interquartile range) if the normality assumption is violated. Categorical variables will be described using the frequency (percentage). The significance level of superiority test will be set at one-sided 0.025 for primary outcome. The significance level will be set at two-sided 0.05 for secondary outcomes. All analyses will be carried out using SAS 9.3 (Cary, NC).

The change from baseline in office SBP at week 12 will be analyzed by a

For peer review only - http://bmjopen.bmj.com/site/about/guidelines.xhtml

mixed-effect model using baseline value as a covariate, treatment group as a fixed effect, and community and interaction between community and treatment as random effects. Multiple imputation will be used for missing data.[28] For the secondary and tertiary outcomes, continuous variables will be compared at all follow-up time points using a mixed-effect model with repeated measurement methods. If there is a normality violation in the continuous variables, a transformation will be performed before the test. Categorical variables will be compared using a generalised linear mixed-effect model. The consistency of treatment effects on the primary outcome will be explored in predefined subgroups, including sex, education, and taking antihypertensive drugs or not and urban or rural community.

Data management and quality control

Data will be collected by using a web-based Research Electronic Data Capture system (https://yu.life.sjtu.edu.cn/redcap/) developed by Shanghai Jiao Tong University. All researchers will receive training about data input, inquiry and modification. Independent data managers will check the system to ensure the integrity, accuracy and timeliness of data. Any modifications of the data can be traced in the Research Electronic Data Capture system. Moreover, patient information will be anonymous, including name, ID number and telephone number, to protect privacy. After the trial is completed, this system will be locked by the data management team. Then the researchers can no longer modify the data. An independent data and safety monitor board will be established to review and interpret the trial data every 6 months.

For peer review only - http://bmjopen.bmj.com/site/about/guidelines.xhtml

Patient and public involvement

The first author (Jian-Feng Tu) and other co-researchers in community health service centers are patients with hypertension. They were involved in the design and conduct of the trial. During the design stage, choice of outcome measures, the number of study visits and methods of recruitment were determined by discussions with patients. Once the trial has been published, a brief summary of the findings will be presented to attendees in the community health service centers.

Ethics and dissemination

This study conforms to the principles of the Declaration of Helsinki. The study protocol (version number: 2.0; June 13 2020) was approved by the ethics committee of Beijing University of Chinese Medicine (No. 2020BZHYLL0104). Meanwhile, written informed consent will be obtained by the clinical research coordinator from all patients before randomization. Results of the trial are expected to be published in a peer-reviewed journal.

Discussion

To our knowledge, this is the first cluster randomised trial to investigate the effectiveness and safety of the smart phone-based TEAS as adjunctive therapy for hypertension.

For peer review only - http://bmjopen.bmj.com/site/about/guidelines.xhtml

This study is a community-based cluster randomised trial. There are several advantages for this design. First, contamination is one of the common biases, but it can be alleviated by the cluster design, because only the communities randomly assigned to the TEAS group will have access to the instrument. Furthermore, communities were chosen with adequate geographical separation from each other. Second, patients will be recruited from their homes, or communities. Unlike hospital-based studies, which only include those who seek medical help, our design will overcome the traditionally low detection of hypertension.

The TEAS in this trial can be received through smart phone at home by the patients themselves. The majority of hypertension patients had to travel by taxi or use public transportation to attend physician visits and prescribe antihypertensive drugs. If the effectiveness of TEAS is verified, this non-pharmaceutical therapy will result in additional savings of cost and burden. The time, expense and lost wages are also identified as barriers for controlling blood pressure. The smart phone-based TEAS at home may contribute to improve adherence to treatment. Furthermore, several patients sometimes forget to take medicine, especially when they are busy working or tripping. As we mentioned before, we could use the background of TEAS software to recognise patients with poor compliance real-timely and remind them to complete treatment.

The mechanism of acupuncture to reducing blood pressure is still unclear. Plentz R

For peer review only - http://bmjopen.bmj.com/site/about/guidelines.xhtml

and colleagues found that TEAS could decrease sympathetic nervous system activity and increase parasympathetic nervous system activity,[29] which may contribute to reducing blood pressure. Moreover, TEAS was shown to have a vasodilator effect.[30] Our animal experiments also indicated that mitogen-activated protein kinases and β-adrenergic receptors were involved in the mechanism of acupuncture's amelioration of hypertension.[31 32]

Our study has several limitations. First, blinding for patients was not feasible; however, several steps will be implemented to minimise potential biases. Outcome assessors will be masked and electronic data capture system with tablet has been developed to separate streams of data collection processes. Moreover, the blood pressure recordings were from automated devices to minimised biases for the key outcome. Second, surrogate outcomes are used in this study. There is good evidence to support improved clinical outcomes with the reductions in blood pressure shown in other trials.[33 34] To show a benefit in clinical events, a much larger and longer trial would be required. Third, patients in the usual care group will be screened for cardiovascular disease risk at baseline, have their blood pressure assessed, and be provided with information about hypertension education. After aware of their elevated blood pressure, some patients in the usual care group might modify their behavior and receiving more treatment than clinical practice. This modification might have reduced the real differences between the groups, which mean that we may underestimate the effectiveness of TEAS.

For peer review only - http://bmjopen.bmj.com/site/about/guidelines.xhtml

This trial may provide high-quality evidence for the effectiveness of the smart phone-based TEAS as adjunctive therapy for hypertension. If the study hypothesis is confirmed, smart phone-based TEAS may help to attain the United Nations General Assembly Action Plan for a one-third reduction in premature mortality from cardiovascular disease.

Trial status

Recruitment was started at 18th November 2020 and is expected to close in June 2022.

For peer review only

For peer review only - http://bmjopen.bmj.com/site/about/guidelines.xhtml

Acknowledgements

Appreciation to every participant in the trial and every personnel in recruitment sites for their contributions.

Contributors

Liu CZ conceived of the study. Liu CZ, Tu JF, and Wang LQ initiated the study design. Yan SY drew up the statistical plan. Kang SB, Yan CQ, Su XT, Shi GX, Mi BH, Lin Y, Wang Y, Li HW, Yang JW, Wang XZ, and Wang X helped with its implementation. Tu JF, and Kang SB drafted the manuscript and all the authors critically revised the manuscript. Liu CZ sought funding. Liu CZ, Tu JF, and Wang LQ sought ethical approval. All authors contributed to the refinement of the study protocol and approved the final manuscript.

Funding

This work was supported by National Key R&D Program of China (grant number: 2019YFC1712100) and Project for Distinguished Young Scholars of Beijing University of Chinese Medicine (grant number: BUCM-2019-JCRC011). The sponsors have no role in the design of the trial, collection, management, analysis, interpretation of data; writing of the report or the decision to submit the report for publication.

Competing interests

None declared.

For peer review only - http://bmjopen.bmj.com/site/about/guidelines.xhtml

Patient consent for publication

Not Applicable.

Ethics approval

Ethical approval for this study was obtained from the Ethics Committee of Beijing University of Chinese Medicine (2020BZHYLL0104).

Data Availability Statement

There are no data in this article.

For peer review only

For peer review only - http://bmjopen.bmj.com/site/about/guidelines.xhtml

REFERENCES

1. GBD 2016 Risk Factors Collaborators. Global, regional, and national comparative risk assessment of 84 behavioural, environmental and occupational, and metabolic risks or clusters of risks, 1990-2016: a systematic analysis for the Global Burden of Disease Study 2016. *Lancet* 2017;390:1345-422.

2. World Health Organize. Hypertension [EB/OL]. https://www.who.int/health-topics/hypertension/, 2021-9-16.

3. Whelton PK, Carey RM, Aronow WS, *et al.* 2017 ACC/AHA/AAPA/ABC/ACPM/AGS/APhA/ASH/ASPC/NMA/PCNA Guideline for the Prevention, Detection, Evaluation, and Management of High Blood Pressure in Adults: A Report of the American College of Cardiology/American Heart Association Task Force on Clinical Practice Guidelines. *Circulation* 2018;138:e484-484e594.

4. Garies S, Hao S, McBrien K, *et al.* Prevalence of Hypertension, Treatment, and Blood Pressure Targets in Canada Associated With the 2017 American College of Cardiology and American Heart Association Blood Pressure Guidelines. *JAMA Netw Open* 2019;2:e190406.

5. Wang JG, Liu L. Global Impact of 2017 American College of Cardiology/American Heart Association Hypertension Guidelines: A Perspective From China. *Circulation* 2018;137:546-8.

6. Venkateshmurthy NS, Geldsetzer P, Jaacks LM, *et al.* Implications of the New American College of Cardiology Guidelines for Hypertension Prevalence in

For peer review only - http://bmjopen.bmj.com/site/about/guidelines.xhtml

India. *JAMA Intern Med* 2018;178:1416-8.

7. Lim SS, Vos T, Flaxman AD*, et al.* A comparative risk assessment of burden of disease and injury attributable to 67 risk factors and risk factor clusters in 21 regions, 1990-2010: a systematic analysis for the Global Burden of Disease Study 2010. *Lancet* 2012;380:2224-60.
8. Writing Group Members, Mozaffarian D, Benjamin EJ*, et al.* Heart Disease and Stroke Statistics-2016 Update: A Report From the American Heart Association. *Circulation* 2016;133:e38-360.
9. Williams B, Mancia G, Spiering W*, et al.* 2018 ESC/ESH Guidelines for the management of arterial hypertension. *Eur Heart J* 2018;39:3021-104.
10. Guidelines for the prevention and treatment of hypertension in China (Revised edition in 2018). Chinese Journal of Cardiovascular Medicine [in Chinese] 2019;24:24-56.
11. Zhao L, Li D, Zheng H*, et al.* Acupuncture as Adjunctive Therapy for Chronic Stable Angina: A Randomized Clinical Trial. *JAMA Intern Med* 2019.
12. Wang Q, Liang D, Wang F*, et al.* Efficacy of electroacupuncture pretreatment for myocardial injury in patients undergoing percutaneous coronary intervention: A randomized clinical trial with a 2-year follow-up. *Int J Cardiol* 2015;194:28-35.
13. Flachskampf FA, Gallasch J, Gefeller O*, et al.* Randomized trial of acupuncture to lower blood pressure. *Circulation* 2007;115:3121-9.
14. Zheng H, Li J, Li Y*, et al.* Acupuncture for patients with mild hypertension: A

For peer review only - http://bmjopen.bmj.com/site/about/guidelines.xhtml

randomized controlled trial. *J Clin Hypertens (Greenwich)* 2019;21:412-20.

15. Yang J, Chen J, Yang M*, et al.* Acupuncture for hypertension. *Cochrane Database Syst Rev* 2018;11:CD008821.

16. Tu JF, Wang LQ, Liu JH*, et al.* Home-based transcutaneous electrical acupoint stimulation for hypertension: a randomized controlled pilot trial. *Hypertens Res* 2021.

17. Chan AW, Tetzlaff JM, Altman DG*, et al.* SPIRIT 2013 statement: defining standard protocol items for clinical trials. *Ann Intern Med* 2013;158:200-7.

18. Wang Y, Shi GX, Tian ZX*, et al.* Transcutaneous electrical acupoint stimulation for high-normal blood pressure: study protocol for a randomized controlled pilot trial. *Trials* 2021;22:140.

19. Jacobsson F, Himmelmann A, Bergbrant A*, et al.* The effect of transcutaneous electric nerve stimulation in patients with therapy-resistant hypertension. *J Hum Hypertens* 2000;14:795-8.

20. Silverdal J, Mourtzinis G, Stener-Victorin E*, et al.* Antihypertensive effect of low-frequency transcutaneous electrical nerve stimulation (TENS) in comparison with drug treatment. *Blood Press* 2012;21:306-10.

21. Climie RE, Schultz MG, Nikolic SB*, et al.* Validity and reliability of central blood pressure estimated by upper arm oscillometric cuff pressure. *Am J Hypertens* 2012;25:414-20.

22. Shimamoto K, Ando K, Fujita T*, et al.* The Japanese Society of Hypertension Guidelines for the Management of Hypertension (JSH 2014). *Hypertens Res*

For peer review only

2014;37:253-390.

23. Wan E, Yu E, Chin WY*, et al.* Evaluation of the responsiveness of Short Form-12 Health Survey version 2 (SF-12v2) in Chinese patients with hypertension in primary care. *Qual Life Res* 2019;28:2851-7.

24. Hallal PC, Victora CG. Reliability and validity of the International Physical Activity Questionnaire (IPAQ). *Med Sci Sports Exerc* 2004;36:556.

25. Yan J, You LM, Yang Q*, et al.* Translation and validation of a Chinese version of the 8-item Morisky medication adherence scale in myocardial infarction patients. *J Eval Clin Pract* 2014;20:311-7.

26. Food and Drug Administration. Principles for clinical evaluation of new antihypertensive drugs [EB/OL]. https://www.fda.gov/media/71363/download, 2021-9-16.

27. Killip S, Mahfoud Z, Pearce K. What is an intracluster correlation coefficient? Crucial concepts for primary care researchers. *Ann Fam Med* 2004;2:204-8.

28. Li P, Stuart EA, Allison DB. Multiple Imputation: A Flexible Tool for Handling Missing Data. *JAMA* 2015;314:1966-7.

29. do Amaral Sartori S, Stein C, Coronel CC*, et al.* Effects of Transcutaneous Electrical Nerve Stimulation in Autonomic Nervous System of Hypertensive Patients: A Randomized Controlled Trial. *Curr Hypertens Rev* 2018;14:66-71.

30. Mannheimer C, Emanuelsson H, Waagstein F. The effect of transcutaneous electrical nerve stimulation (TENS) on catecholamine metabolism during pacing-induced angina pectoris and the influence of naloxone. *Pain*

For peer review only - http://bmjopen.bmj.com/site/about/guidelines.xhtml

For peer review only

1990;41:27-34.

31. Wang XR, Yang JW, Ji CS, *et al.* Inhibition of NADPH Oxidase-Dependent Oxidative Stress in the Rostral Ventrolateral Medulla Mediates the Antihypertensive Effects of Acupuncture in Spontaneously Hypertensive Rats. *Hypertension* 2018;71:356-65.

32. Yang JW, Ye Y, Wang XR, *et al.* Acupuncture Attenuates Renal Sympathetic Activity and Blood Pressure via Beta-Adrenergic Receptors in Spontaneously Hypertensive Rats. *Neural Plast* 2017;2017:8696402.

33. Roshandel G, Khoshnia M, Poustchi H, *et al.* Effectiveness of polypill for primary and secondary prevention of cardiovascular diseases (PolyIran): a pragmatic, cluster-randomised trial. *Lancet* 2019;394:672-83.

34. Yusuf S, Lonn E, Pais P, *et al.* Blood-Pressure and Cholesterol Lowering in Persons without Cardiovascular Disease. *N Engl J Med* 2016;374:2032-43.

For peer review only - http://bmjopen.bmj.com/site/about/guidelines.xhtml

Table 1 Locations of acupoints in the TEAS group

Acupoints	Locations
Taichong (LR3)	On the dorsum of the foot, between the first and second metatarsal bones, in the depression distal to the junction of the bases of the two bones, over the dorsalis pedis artery.
Zusanli (ST36)	On the anterior aspect of the leg, on the line connecting ST35 with ST41, 3 cun inferior to ST35.
Hegu (LI4)	On the dorsum of the hand, radial to the midpoint of the second metacarpal bone.
Quchi (LI11)	On the lateral aspect of the elbow, at the midpoint of the line connecting LU5 with the lateral epicondyle of the humerus.

[a] 1 cun (≈20 mm) is defined as the width of the interphalangeal joint of patient's thumb.

For peer review only

For peer review only - http://bmjopen.bmj.com/site/about/guidelines.xhtml

Figure legends

Figure 1 Flow diagram

TEAS, transcutaneous electrical acupoint stimulation.

Figure 2 Locations of acupoints

Figure 3 Schedule of enrollment, intervention and assessments

TEAS, transcutaneous electrical acupoint stimulation.

For peer review only

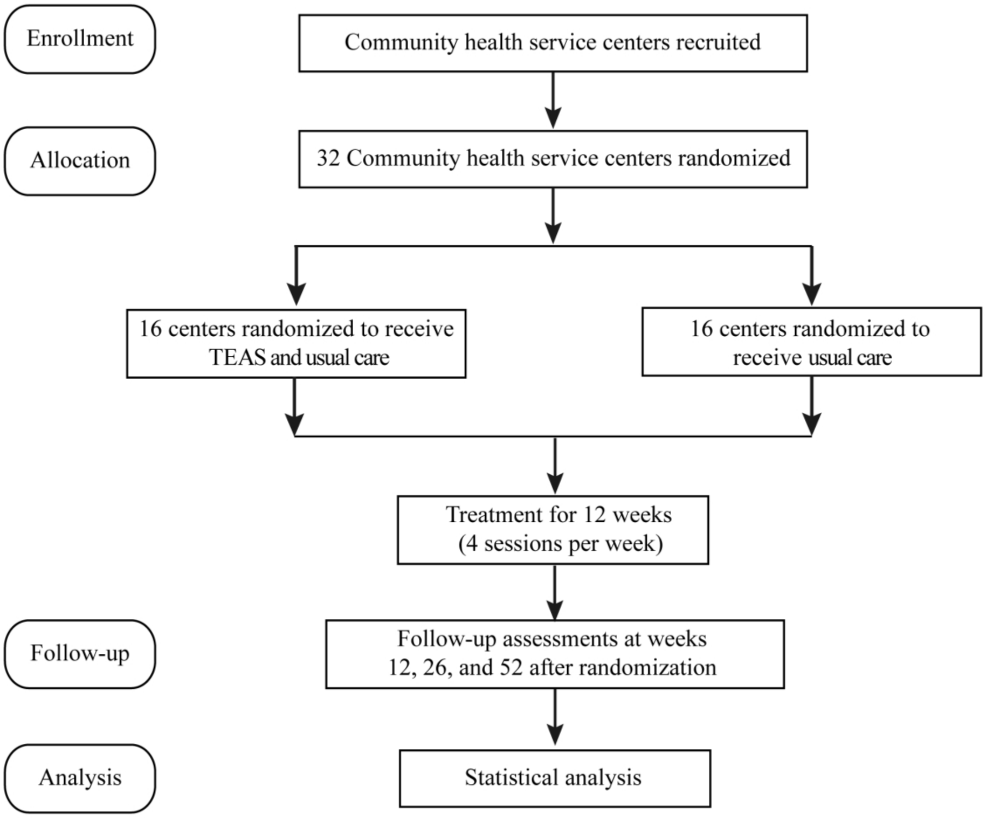

Figure 1 Flow diagram
TEAS, transcutaneous electrical acupoint stimulation.

177x147mm (300 x 300 DPI)

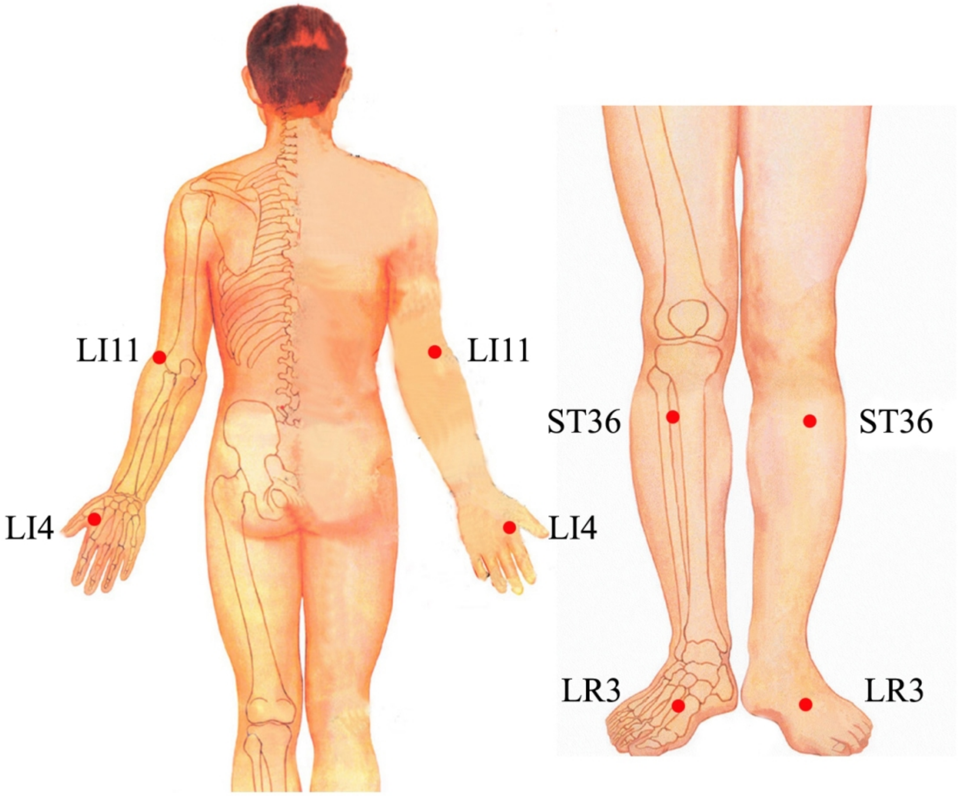

Figure 2 Locations of acupoints

122x99mm (300 x 300 DPI)

	STUDY PERIOD				
	Enrolment	Allocation	Post-allocation		Closeout
TIME POINT	Week -1	Week 0	Week 12	Week 26	Week 52
ENROLLMENT:					
[Eligibility screen]	×				
[Informed consent]	×				
[Randomization]		×			
INTERVENTION:					
[TEAS+usual care]		◆——	——◆		
[usual care]		◆——	——◆		
ASSESSMENTS:					
[systolic blood pressure]		×	×	×	×
[diastolic blood pressure]		×	×	×	×
[blood pressure control rate]		×	×	×	×
[proportion of patients taking antihypertensive drugs]		×	×	×	×
[number of antihypertensive drugs]		×	×	×	×
[12-Item Short-Form]		×	×	×	×
[body mass index]		×	×	×	×
[waist circumference]		×	×	×	×
[physical activity]		×	×	×	×
[medication adherence]		×	×	×	×
[blinding test]			×		
[serious adverse events]			×	×	×
[clinical events]			×	×	×

Figure 3 Schedule of enrollment, intervention and assessments
TEAS, transcutaneous electrical acupoint stimulation.

188x156mm (300 x 300 DPI)

eTable 1 The list of Community Health Service Centers

Number	Institution	District
1	Jianguomen Community Health Service Center	Dongcheng District
2	Hepingli Community Health Service Center	Dongcheng District
3	Shichahai Community Health Service Center	Xicheng District
4	Yuetan Community Health Service Center	Xicheng District
5	Sunhe Community Health Service Center	Chaoyang District
6	Cuigezhuang Second Community Health Service Center	Chaoyang District
7	Nanyuan Community Health Service Center	Fengtai District
8	Dahongmen Community Health Service Center	Fengtai District
9	Jindingjie Community Health Service Center	Shijingshan District
10	Guangning Street Community Health Service Center	Shijingshan District
11	Qinglongqiao Community Health Service Center	Haidian District
12	Malanwa Community Health Service Station	Haidian District
13	Tianzhu Community Health Service Center	Shunyi District
14	Zhaoquanying Community Health Service Center	Shunyi District
15	Yongshun Community Health Service Center	Tongzhou District
16	Taihu Community Health Service Center	Tongzhou District
17	Xihongmen Community Health Service Center	Daxing District
18	Changziying Community Health Service Center	Daxing District
19	Liangxiang Community Health Service Station	Fangshan District
20	Changyang Community Health Service Center	Fangshan District

For peer review only

21	Junzhuang Community Health Service Center	Mentougou District
22	Zaitang Community Health Service Center	Mentougou District
23	Shahe Community Health Service Center	Changping District
24	Huilongguan Community Health Service Center	Changping District
25	Huangsongyu Community Health Service Center	Pinggu District
26	Yukou Community Health Service Center	Pinggu District
27	Henanzhai Community Health Service Center	Miyun District
28	Mujianyu Community Health Service Center	Miyun District
29	Yangsong Community Health Service Center	Huairou District
30	Miaocheng Community Health Service Center	Huairou District
31	Zhangshanying Community Health Service Center	Yanqing District
32	Yongning Community Health Service Center	Yanqing District

For peer review only

SPIRIT

STANDARD PROTOCOL ITEMS: RECOMMENDATIONS FOR INTERVENTIONAL TRIALS

SPIRIT 2013 Checklist: Recommended items to address in a clinical trial protocol and related documents*

For peer review only

Section/item	Item No	Description	Page
**Administrative information**			
Title	1	Descriptive title identifying the study design, population, interventions, and, if applicable, trial acronym	1
Trial registration	2a	Trial identifier and registry name. If not yet registered, name of intended registry	4
	2b	All items from the World Health Organization Trial Registration Data Set	7-13
Protocol version	3	Date and version identifier	7,16
Funding	4	Sources and types of financial, material, and other support	20
Roles and responsibilities	5a	Names, affiliations, and roles of protocol contributors	1,20
	5b	Name and contact information for the trial sponsor	20
	5c	Role of study sponsor and funders, if any, in study design; collection, management, analysis, and interpretation of data; writing of the report; and the decision to submit the report for publication, including whether they will have ultimate authority over any of these activities	20
	5d	Composition, roles, and responsibilities of the coordinating centre, steering committee, endpoint adjudication committee, data management team, and other individuals or groups overseeing the trial, if applicable (see Item 21a for data monitoring committee)	15

SPIRIT 2013 Checklist: Recommended items to address in a clinical trial protocol and related documents*(continued)

Section/item	Item No	Description	Page
**Introduction**			
Background and rationale	6a	Description of research question and justification for undertaking the trial, including summary of relevant studies (published and unpublished) examining benefits and harms for each intervention	6-7
	6b	Explanation for choice of comparators	6-7
Objectives	7	Specific objectives or hypotheses	7
Trial design	8	Description of trial design including type of trial (eg, parallel group, crossover, factorial, single group), allocation ratio, and framework (eg, superiority, equivalence, noninferiority, exploratory)	7
**Methods: Participants, interventions, and outcomes**			
Study setting	9	Description of study settings (eg, community clinic, academic hospital) and list of countries where data will be collected. Reference to where list of study sites can be obtained	7,Supplement 1
Eligibility criteria	10	Inclusion and exclusion criteria for participants. If applicable, eligibility criteria for study centres and individuals who will perform the interventions (eg, surgeons, psychotherapists)	8-9
Interventions	11a	Interventions for each group with sufficient detail to allow replication, including how and when they will be administered	10-11
	11b	Criteria for discontinuing or modifying allocated interventions for a given trial participant (eg, drug dose change in response to harms, participant request, or improving/worsening disease)	10

For peer review only - http://bmjopen.bmj.com/site/about/guidelines.xhtml

SPIRIT 2013 Checklist: Recommended items to address in a clinical trial protocol and related documents*(continued)

Section/item	Item No	Description	Page
	11c	Strategies to improve adherence to intervention protocols, and any procedures for monitoring adherence (eg, drug tablet return, laboratory tests)	11
	11d	Relevant concomitant care and interventions that are permitted or prohibited during the trial	10
Outcomes	12	Primary, secondary, and other outcomes, including the specific measurement variable (eg, systolic blood pressure), analysis metric (eg, change from baseline, final value, time to event), method of aggregation (eg, median, proportion), and time point for each outcome. Explanation of the clinical relevance of chosen efficacy and harm outcomes is strongly recommended	12-13
Participant timeline	13	Time schedule of enrolment, interventions (including any run-ins and washouts), assessments, and visits for participants. A schematic diagram is highly recommended (see Figure)	7, Figure 1 and 3
Sample size	14	Estimated number of participants needed to achieve study objectives and how it was determined, including clinical and statistical assumptions supporting any sample size calculations	13
Recruitment	15	Strategies for achieving adequate participant enrolment to reach target sample size	7-8
**Methods: Assignment of interventions (for controlled trials)**			
Allocation:			
Sequence generation	16a	Method of generating the allocation sequence (eg, computer-generated random numbers), and list of any factors for stratification. To reduce predictability of a random sequence, details of any planned restriction (eg, blocking) should be provided in a separate document that is unavailable to those who enrol participants or assign interventions	9

SPIRIT 2013 Checklist: Recommended items to address in a clinical trial protocol and related documents*(continued)

Section/item	Item No	Description	Page
Allocation concealment mechanism	16b	Mechanism of implementing the allocation sequence (eg, central telephone; sequentially numbered, opaque, sealed envelopes), describing any steps to conceal the sequence until interventions are assigned	9
Implementation	16c	Who will generate the allocation sequence, who will enrol participants, and who will assign participants to interventions	9
Blinding (masking)	17a	Who will be blinded after assignment to interventions (eg, trial participants, care providers, outcome assessors, data analysts), and how	9
	17b	If blinded, circumstances under which unblinding is permissible, and procedure for revealing a participant's allocated intervention during the trial	9
**Methods: Data collection, management, and analysis**			
Data collection methods	18a	Plans for assessment and collection of outcome, baseline, and other trial data, including any related processes to promote data quality (eg, duplicate measurements, training of assessors) and a description of study instruments (eg, questionnaires, laboratory tests) along with their reliability and validity, if known. Reference to where data collection forms can be found, if not in the protocol	12-13
	18b	Plans to promote participant retention and complete follow-up, including list of any outcome data to be collected for participants who discontinue or deviate from intervention protocols	12
Data management	19	Plans for data entry, coding, security, and storage, including any related processes to promote data quality (eg, double data entry; range checks for data values). Reference to where details of data management procedures can be found, if not in the protocol	15
Statistical methods	20a	Statistical methods for analysing primary and secondary outcomes. Reference to where other details of the statistical analysis plan can be found, if not in the protocol	14-15

SPIRIT 2013 Checklist: Recommended items to address in a clinical trial protocol and related documents*(continued)

Section/item	Item No	Description	Page
	20b	Methods for any additional analyses (eg, subgroup and adjusted analyses)	14-15
	20c	Definition of analysis population relating to protocol non-adherence (eg, as randomised analysis), and any statistical methods to handle missing data (eg, multiple imputation)	14-15
**Methods: Monitoring**			
Data monitoring	21a	Composition of data monitoring committee (DMC); summary of its role and reporting structure; statement of whether it is independent from the sponsor and competing interests; and reference to where further details about its charter can be found, if not in the protocol. Alternatively, an explanation of why a DMC is not needed	15
	21b	Description of any interim analyses and stopping guidelines, including who will have access to these interim results and make the final decision to terminate the trial	Not applicable
Harms	22	Plans for collecting, assessing, reporting, and managing solicited and spontaneously reported adverse events and other unintended effects of trial interventions or trial conduct	13
Auditing	23	Frequency and procedures for auditing trial conduct, if any, and whether the process will be independent from investigators and the sponsor	15
**Ethics and dissemination**			
Research ethics approval	24	Plans for seeking research ethics committee/institutional review board (REC/IRB) approval	16
Protocol amendments	25	Plans for communicating important protocol modifications (eg, changes to eligibility criteria, outcomes, analyses) to relevant parties (eg, investigators, REC/IRBs, trial participants, trial registries, journals, regulators)	Not applicable

For peer review only - http://bmjopen.bmj.com/site/about/guidelines.xhtml

SPIRIT 2013 Checklist: Recommended items to address in a clinical trial protocol and related documents*(continued)

Section/item	Item No	Description	Page
Consent or assent	26a	Who will obtain informed consent or assent from potential trial participants or authorised surrogates, and how (see Item 32)	16
	26b	Additional consent provisions for collection and use of participant data and biological specimens in ancillary studies, if applicable	Not applicable
Confidentiality	27	How personal information about potential and enrolled participants will be collected, shared, and maintained in order to protect confidentiality before, during, and after the trial	15
Declaration of interests	28	Financial and other competing interests for principal investigators for the overall trial and each study site	20
Access to data	29	Statement of who will have access to the final trial dataset, and disclosure of contractual agreements that limit such access for investigators	21
Ancillary and post-trial care	30	Provisions, if any, for ancillary and post-trial care, and for compensation to those who suffer harm from trial participation	Not applicable
Dissemination policy	31a	Plans for investigators and sponsor to communicate trial results to participants, healthcare professionals, the public, and other relevant groups (eg, via publication, reporting in results databases, or other data sharing arrangements), including any publication restrictions	16
	31b	Authorship eligibility guidelines and any intended use of professional writers	20
	31c	Plans, if any, for granting public access to the full protocol, participant-level dataset, and statistical code	Not applicable

For peer review only - http://bmjopen.bmj.com/site/about/guidelines.xhtml

SPIRIT 2013 Checklist: Recommended items to address in a clinical trial protocol and related documents*(continued)

Section/item	Item No	Description	Page
**Appendices**			
Informed consent materials	32	Model consent form and other related documentation given to participants and authorised surrogates	Not applicable
Biological specimens	33	Plans for collection, laboratory evaluation, and storage of biological specimens for genetic or molecular analysis in the current trial and for future use in ancillary studies, if applicable	Not applicable

*It is strongly recommended that this checklist be read in conjunction with the SPIRIT 2013 Explanation & Elaboration for important clarification on the items. Amendments to the protocol should be tracked and dated. The SPIRIT checklist is copyrighted by the SPIRIT Group under the Creative Commons "Attribution-NonCommercial-NoDerivs 3.0 Unported" license.

For peer review only

BMJ Open

Smart phone-based Transcutaneous electrical acupoint stimulation as Adjunctive Therapy for Hypertension (STAT-H trial): protocol for a cluster randomised controlled trial

Journal:	*BMJ Open*
Manuscript ID	bmjopen-2021-058172.R1
Article Type:	Protocol
Date Submitted by the Author:	29-Mar-2022
Complete List of Authors:	Tu, Jian Feng; Beijing University of Chinese Medicine, International Acupuncture and Moxibustion Innovation Institute, School of Acupuncture-Moxibustion and Tuina<br>Kang, Si-Bo; Beijing University of Chinese Medicine, International Acupuncture and Moxibustion Innovation Institute, School of Acupuncture-Moxibustion and Tuina<br>Wang, Li-Qiong; Beijing University of Chinese Medicine, International Acupuncture and Moxibustion Innovation Institute, School of Acupuncture-Moxibustion and Tuina<br>YAN, Shiyan; Beijing University of Chinese Medicine, International Acupuncture and Moxibustion Innovation Institute, School of Acupuncture-Moxibustion and Tuina<br>Yan, Chao-qun; Dongzhimen Hospital, Beijing University of Chinese Medicine, Department of Acupuncture and Moxibustion<br>Su, Xin-Tong; Beijing University of Chinese Medicine, International Acupuncture and Moxibustion Innovation Institute, School of Acupuncture-Moxibustion and Tuina<br>Shi, Guangxia; Beijing University of Chinese Medicine, International Acupuncture and Moxibustion Innovation Institute, School of Acupuncture-Moxibustion and Tuina<br>Mi, Bao-Hong; Beijing University of Chinese Medicine, International Acupuncture and Moxibustion Innovation Institute, School of Acupuncture-Moxibustion and Tuina<br>Lin, Ying; Beijing University of Chinese Medicine, International Acupuncture and Moxibustion Innovation Institute, School of Acupuncture-Moxibustion and Tuina<br>Wang, Yu; Beijing University of Chinese Medicine, International Acupuncture and Moxibustion Innovation Institute, School of Acupuncture-Moxibustion and Tuina<br>Li, He-Wen; Beijing University of Chinese Medicine, International Acupuncture and Moxibustion Innovation Institute, School of Acupuncture-Moxibustion and Tuina<br>Wang, Xue-Zhou; Beijing University of Chinese Medicine, International Acupuncture and Moxibustion Innovation Institute, School of Acupuncture-Moxibustion and Tuina<br>Wang, Xiao; Beijing University of Chinese Medicine, International Acupuncture and Moxibustion Innovation Institute, School of Acupuncture-Moxibustion and Tuina<br>Yang, Jing-Wen; Beijing University of Chinese Medicine, International

	Acupuncture and Moxibustion Innovation Institute, School of Acupuncture-Moxibustion and Tuina<br>Liu, Cun-Zhi; Beijing University of Chinese Medicine, International Acupuncture and Moxibustion Innovation Institute, School of Acupuncture-Moxibustion and Tuina
**Primary Subject Heading**:	Complementary medicine
Secondary Subject Heading:	Cardiovascular medicine
Keywords:	COMPLEMENTARY MEDICINE, Hypertension < CARDIOLOGY, Clinical trials < THERAPEUTICS

SCHOLARONE™
Manuscripts

Smart phone-based Transcutaneous electrical acupoint stimulation as Adjunctive Therapy for Hypertension (STAT-H trial): protocol for a cluster randomised controlled trial

Jian-Feng Tu[1, #], Si-Bo Kang[1, #], Li-Qiong Wang[1], Shi-Yan Yan[1], Chao-Qun Yan[2], Xin-Tong Su[1], Guang-Xia Shi[1], Bao-Hong Mi[1], Ying Lin[1], Yu Wang[1], He-Wen Li[1], Xue-Zhou Wang[1], Xiao Wang[1], Jing-Wen Yang[1], Cun-Zhi Liu[1, *]

1 International Acupuncture and Moxibustion Innovation Institute, School of Acupuncture-Moxibustion and Tuina, Beijing University of Chinese Medicine, Beijing, China

2 Department of Acupuncture and Moxibustion, Dongzhimen Hospital, Beijing University of Chinese Medicine, Beijing, China

[#] The two authors contributed equally.

*Corresponding author: Cun-Zhi Liu

International Acupuncture and Moxibustion Innovation Institute, School of Acupuncture-Moxibustion and Tuina, Beijing University of Chinese Medicine, No. 11, Bei San Huan Dong Lu, Chaoyang District, Beijing 100029, China.

E-mail address: lcz_tg@126.com

Running title: Smart phone-based TEAS as Adjunctive Therapy for Hypertension

Disclaimers: The authors have no conflicts of interest to declare.

For peer review only - http://bmjopen.bmj.com/site/about/guidelines.xhtml

Word count: 2939 words

Number of figures: 3

Number of tables: 1

ORCID

Cun-Zhi Liu https://orcid.org/0000-0001-8031-5667

Jian-Feng Tu http://orcid.org/0000-0003-2887-3383

For peer review only

For peer review only - http://bmjopen.bmj.com/site/about/guidelines.xhtml

ABSTRACT

Introduction: Hypertension is a common risk factor for cardiovascular disease. Transcutaneous electrical acupoint stimulation (TEAS) may be effective for hypertension, but the evidence remains limited. The aim of this study is to evaluate the effectiveness and safety of the smart phone-based TEAS as adjunctive therapy for hypertension.

Methods and analysis: This study is a 52-week cluster randomised controlled trial with 1600 hypertension patients in 32 community health service centers. Patients who meet the inclusion criteria will be randomised into usual care group or TEAS group in a 1:1 ratio. All patients will be provided with usual care as recommended by the guidelines. In addition to this, patients in the TEAS group will receive non-invasive acupoint electrical stimulation for 30 minutes at home, 4 times weekly for 12 weeks. The primary outcome will be the mean difference in the changes in office systolic blood pressure from baseline to 12 weeks between TEAS and usual care groups. Secondary outcomes will include the change of mean diastolic blood pressure, proportion of patients with controlled blood pressure (blood pressure ＜ 140/90 mmHg), proportion of patients taking antihypertensive drugs, change in number of antihypertensive drugs, and changes in 12-Item Short-Form. Tertiary outcomes will include change in body mass index, change in waist circumference, physical activity, and medication adherence. Safety outcomes will be any serious adverse events and clinical events.

Ethics and dissemination: This study has been approved by ethics committee of Beijing University of Chinese Medicine (No. 2020BZHYLL0104). Written informed

For peer review only - http://bmjopen.bmj.com/site/about/guidelines.xhtml

For peer review only

consent will be obtained from all patients before randomisation. Trial results will be disseminated in peer-reviewed publications.

Trial registration: ChiCTR2000039400.

Keywords: Transcutaneous electrical acupoint stimulation; hypertension; usual care; cluster randomised controlled trial; protocol

For peer review only

For peer review only - http://bmjopen.bmj.com/site/about/guidelines.xhtml

Strengths and limitations of this study

- In this randomised controlled trial, a large population of 1600 hypertension patients will be recruited from 32 community health service centers.
- This community-based cluster trial could lower the risk of contamination as much as possible and overcome the traditionally low detection of hypertension in hospital-based studies.
- The smart phone-based TEAS can be received at home by the patients themselves to reduce the burden of logistics and lost wages.
- The background of TEAS software would recognise patients with poor compliance real-timely and remind them of receiving TEAS to improve the adherence rate.
- Surrogate outcome, not clinical event, is used as primary outcome and blinding for patients was not feasible in this study.

For peer review only

For peer review only - http://bmjopen.bmj.com/site/about/guidelines.xhtml

INTRODUCTION

Cardiovascular disease is the most common cause of premature death and disability worldwide, and hypertension is the most common risk factor for cardiovascular disease.[1] World Health Organization (WHO) suggested that the global prevalence of hypertension was 20% for females and 25% for males.[2] Moreover, according to the latest American College of Cardiology/American Heart Association (ACC/AHA) guideline,[3] the prevalence of hypertension increased to nearly 50%.[4-6] Hypertension accounted for 9.4 million deaths and 7.0% of global disability-adjusted life-years (DALYs).[7] The total costs of hypertension will increase to an estimated $274 billion by 2030.[8]

Lifestyle modifications are recommended by all guidelines in different countries,[3 9 10] while most patients are unable to sustain lifestyle changes. Antihypertensive drugs are widely prescribed for patients with hypertension. However, fewer than 20% of patients with hypertension have their blood pressure controlled.[10] The reasons for low control rate of blood pressure include the poor adherence of medicine and the burden of logistics. Identifying non-pharmaceutical therapy with good adherence that can be done at home is urgently needed to reduce blood pressure in patients with hypertension.

Acupuncture, as a non-pharmaceutical therapy, was used for several cardiovascular diseases.[11 12] Previous studies indicated that acupuncture could decrease blood pressure[13 14] and a recent systematic review found that acupuncture as adjunctive

For peer review only - http://bmjopen.bmj.com/site/about/guidelines.xhtml

therapy may be effective for hypertension.[15] However, acupuncture also has the same limitation of logistical burdens as the antihypertensive drugs. Transcutaneous electrical acupoint stimulation (TEAS), a non-invasive acupuncture therapy, has a similar effect with acupuncture[16-18] and can be performed at home by the patients themselves. Our pilot study found that TEAS added to usual care for patients with hypertension was feasible and may be a potential treatment option.[19] The aim of this cluster randomised controlled trial is to evaluate the effectiveness and safety of the smart phone-based TEAS as adjunctive therapy for hypertension.

METHODS

Study Design

This two-arm, open-label, cluster randomised controlled trial will be conducted at 32 community health service centers in China. The total trial period was 52 weeks, including 12 weeks of treatment and 40 weeks of follow-up. The study protocol (version number: 2.0; June 13, 2020) has been registered on Chinese clinical trial registry (No. ChiCTR2000039400). The protocol will be reported following the SPIRIT guideline.[20] Figure 1 shows the flow diagram of the trial.

Study recruitment

There are 16 districts and 346 community health service centers in Beijing, China. Of them, 32 community health service centers (Supplement 1), 2 centers in each district, will be recruited through *Beijing Administration of Traditional Chinese Medicine* and

For peer review only - http://bmjopen.bmj.com/site/about/guidelines.xhtml

Beijing Association of Acupuncture-Moxibustion. Patients will be diagnosed as hypertension according to the European guideline[9] and Chinese guideline[10]. About 50 patients will be enrolled in each center by their family doctors. The family doctors will conduct a preliminary screening to exclude ineligible patients, then the office blood pressure will be measured in the consulting room by clinical research coordinators to determine whether the patients will be finally included or not.

Participants

Inclusion criteria:

1. Office systolic blood pressure (SBP) from 140 to 159 mm Hg and/or diastolic blood pressure (DBP) from 90 to 99 mm Hg for patients who did not take antihypertensive drugs previously, or Office SBP from 120 to 159 mm Hg and/or DBP from 80 to 99 mm Hg for patients who did not change antihypertensive drugs at least 1 month

2. Aged between 18 and 65 years (male or female)

3. Answering and completing the questionnaires

4. Using smart phone

5. Written informed consent

Exclusion criteria:

1. Other diseases that can affect the blood pressure, including but not limited to diseases of renal parenchyma, renal artery stenosis, obstructive sleep apnea syndrome, and primary hyperaldosteronism

2. Receiving medicines that affect blood pressure except antihypertensive drugs in last

For peer review only - http://bmjopen.bmj.com/site/about/guidelines.xhtml

month, including but not limited to glucocorticoids and central nervous system inhibitors

3. Contraindications for the use of acupoint stimulators: wearing a pacemaker and other implanted medical devices; epilepsy; scars, bruises, scratches or inflammation on the skin of the acupoints; allergic to electrodes

4. Uncontrolled diabetes (HbA1c $\geqslant$ 6.5%)

5. Drug or alcohol abuse

6. Pregnant or breastfeeding

7. Participated in other clinical trials in the past 3 months

Randomization and masking

The unit of randomization will be the community health service centers. Thirty-two centers will be randomised to either TEAS or usual care group in a 1:1 ratio with a central randomization system. All patients within the defined center will receive the same allocated treatment. Each center will not be randomised until total 50 patients are recruited to avoid selection bias. The randomization sequence will be generated by an independent statistician with the Stata 12.0 software and will be stratified by districts. The patients and the acupuncturists who teach patients how to locate acupoints and to receive TEAS at home will not be blinded. However, the family doctors who prescribe antihypertensive drugs, outcome assessors and statisticians will be blinded.

Interventions

For peer review only - http://bmjopen.bmj.com/site/about/guidelines.xhtml

Usual care group

All patients will be provided usual care at the respective community health service center, in conjunction with any ongoing care that they are receiving from their family doctor. Patients at high or very high cardiovascular risk will receive antihypertensive drugs according to the recommendation of the guidelines,[9 10] including diuretics, β-blockers, calcium channel blockers, angiotensin receptor blockers, and angiotensin-converting enzyme inhibitors. The choice of antihypertensive drugs will be individualised, with consideration of patients' coexisting conditions and adverse effects to the medications. Research team will engage in consistent communication with each patient's family doctor, including a standard letter conveying the reminder that the doctor is free to implement any additional therapies that are deemed to be appropriate, including increase or decrease the type and dosage of antihypertensive drugs. Research team will also send education materials to all patients. Content will include information about hypertension, its clinical features, epidemiology, diagnostic criteria, and treatment options, as well as the role and suggestions of lifestyle intervention in hypertension.

TEAS group

In addition to the usual care, the patients in the TEAS group will receive 48 sessions of TEAS treatment (4 times weekly for 12 weeks). TEAS will be performed using a portable instrument (SDP-310, Hwato, Suzhou Medical Appliances Co, Ltd., Suzhou, China) with a pair of rounded electrodes (diameter = 38 mm) through smart phone. The

For peer review only - http://bmjopen.bmj.com/site/about/guidelines.xhtml

adhesive electrodes will be applied to the skin as illustrated in Figure 2 after the skin sites are cleaned with alcohol to avoid any barrier conduction of the electrical current. The instrument has ten levels of stimulus intensities, and patients asked to increase the stimulus intensity until local, rhythmic contractions of the muscle are obtained without producing pain. According to the previous studies,[16 19 21 22] bilateral *Hegu* (LI4), *Quchi* (LI11), *Zusanli* (ST36), and *Taichong* (LR3) will be used in our trial. On the first day, the electrodes will be placed at the ipsilateral LI4 and LI11, and the appropriate stimulus intensity will last for 15 minutes. Then the opposite arm will be stimulated in the same way for another 15 minutes. On the third day, the ST36 and LR3 will be stimulated for 15 minutes per leg in the same way. Two pairs of acupoints (LI4-LI11 and ST36-LR3) will be stimulated alternatively every other day. Locations of acupoints are shown in Table 1. The acupuncturists will train the patients on how to locate the acupoints and use the instrument face-to-face. Instructions and acupoint maps will also be provided to help patients to locate the acupoints at home. The patients will be told that the acupoints stimulation can be done when they are leisure at home, like reading the newspaper or watching TV. The date, duration, and specific acupoints of each acupoint stimuli will be recorded real-timely by TEAS software. Thus, the investigator could recognise patients with poor compliance in a timely manner and prompt them to complete the predefined 48 sessions.

Outcomes

The primary outcome will be the mean difference in the changes in office SBP from

For peer review only - http://bmjopen.bmj.com/site/about/guidelines.xhtml

baseline to 12 weeks between TEAS and usual care groups. SBP will be measured as per the WHO STEPS protocol to ensure consistency of recordings, and an automated monitor (HEM-7136, Omron Corporation, Kyoto, Japan) will be used at each visit. Patients will be asked to rest quietly for at least 5 minutes in the sitting position before measuring blood pressure. Blood pressure of both arms will be measured at baseline with the upper arm at heart level, and the arm with higher blood pressure will be the one measured throughout the entire study. At each time point, the blood pressure measurement will be repeated 3 times every 5 minutes, and the last two will be averaged.[23 24]

Secondary outcomes will include differences in the changes in office DBP between the TEAS and usual care groups; the proportion of patients with well-controlled blood pressure (office blood pressure < 140/90 mmHg); the proportion of patients taking antihypertensive drugs; differences in the changes in number of antihypertensive drugs; and differences in the changes in 12-Item Short-Form (SF-12).[25] Tertiary outcomes will include change in body mass index (BMI); change in waist circumference; physical activity assessed using International Physical Activity Questionnaire (IPAQ);[26] medication adherence assessed using the 8-item Morisky Medication Adherence Scale 8 (MMAS-8)[27] in patients taking antihypertensive drugs in both groups; and TEAS adherence assessed using number of sessions completed in TEAS group. All secondary outcomes and tertiary outcomes will be measured at weeks 12, 26 and 52. Moreover, blinding test of outcome assessors will be measured at week 12.

For peer review only - http://bmjopen.bmj.com/site/about/guidelines.xhtml

Safety outcomes will be any serious adverse events and clinical events (death, myocardial infarction, stroke, and cardiovascular-related hospitalisations) at weeks 12, 26 and 52 in both groups. The schedule of enrolment, intervention and assessments is shown in Figure 3.

Sample size calculation

The effective sample size (ESS) of 392 patients in each group is calculated for SBP on the basis of an alpha level of 0.05 (two-sided), a beta level of 0.80, an expected between-group difference of 6 mm Hg,[19] a standard deviation of 10 mm Hg, and a superiority margin of 4 mm Hg,[28] if randomization takes place at the individual level. With average 40 patients per community and an intra-cluster correlation (ICC) of 0.01,[29] the ESS should be multiplied by a design effect of 1.39, leading to 560 patients in 14 communities per group (1120 patients in 28 communities in total). As our experience suggests that up to 10% of the randomised communities and up to 20% of the recruited patients may drop out of the study, we aim to recruit 32 communities (16 per group) including 50 patients each, resulting in a target sample size of 1600 recruited patients in total (800 per group).

Statistical analysis

A full analysis set, a per-protocol set, and a safety set will be used in this trial. The full analysis set will consist of all randomised patients according to the intention-to-treat

For peer review only - http://bmjopen.bmj.com/site/about/guidelines.xhtml

principle. The full analysis set will be the primary analysis, and all analyses will be conducted for this population if not otherwise stated. The per-protocol set will include those who complete all follow-ups on time and without major violations. Major violations include but are not limited to not ineligibility according to the inclusion and exclusion criteria, receiving medicines that affect blood pressure except antihypertensive drugs, or not completing ⩽ 38 sessions TEAS (80% of prespecified 48 sessions). The per-protocol set will be the secondary analysis set and will be used for the sensitivity analyses for the primary outcome. All those who receive at least one session of TEAS will be defined as the safety set, which is used for the safety analyses.

Baseline characteristics will be summarised by groups. Continuous variables will be described using the mean (standard deviation), or the median (interquartile range) if the normality assumption is violated. Categorical variables will be described using the frequency (percentage). The significance level of superiority test will be set at one-sided 0.025 for the primary outcome. The significance level will be set at two-sided 0.05 for secondary outcomes. All analyses will be carried out using SAS 9.3 (Cary, NC).

The change from baseline in office SBP at week 12 will be analyzed by a mixed-effect model using baseline value as a covariate, treatment group as a fixed effect, and community and interaction between community and treatment as random effects. Multiple imputation will be used for missing data.[30] For the secondary and tertiary outcomes, continuous variables will be compared at all follow-up time points using a

For peer review only - http://bmjopen.bmj.com/site/about/guidelines.xhtml

mixed-effect model with repeated measurement methods. If there is a normality violation in the continuous variables, a transformation will be performed before the test. Categorical variables will be compared using a generalised linear mixed-effect model. The consistency of treatment effects on the primary outcome will be explored in predefined subgroups, including sex, education, and taking antihypertensive drugs or not and urban or rural community.

Data management and quality control

Data will be collected by using a web-based Research Electronic Data Capture system (https://yu.life.sjtu.edu.cn/redcap/) developed by Shanghai Jiao Tong University. All researchers will receive training about data input, inquiry and modification. Independent data managers will check the system to ensure the integrity, accuracy and timeliness of data. Any modifications of the data can be traced in the Research Electronic Data Capture system. Moreover, patient information will be anonymous, including name, ID number and telephone number, to protect privacy. After the trial is completed, this system will be locked by the data management team. Then the researchers can no longer modify the data. An independent data and safety monitor board will be established to review and interpret the trial data every 6 months.

Patient and public involvement

The first author (Jian-Feng Tu) and other co-researchers in community health service centers are patients with hypertension. They were involved in the design and conduct

For peer review only - http://bmjopen.bmj.com/site/about/guidelines.xhtml

of the trial. During the design stage, choice of outcome measures, the number of study visits and methods of recruitment were determined by discussions with patients. Once the trial has been published, a brief summary of the findings will be presented to attendees in the community health service centers.

Ethics and dissemination

This study conforms to the principles of the Declaration of Helsinki. The study protocol (version number: 2.0; June 13, 2020) was approved by the ethics committee of Beijing University of Chinese Medicine (No. 2020BZHYLL0104). Meanwhile, written informed consent will be obtained by the clinical research coordinator from all patients before randomization. Results of the trial are expected to be published in a peer-reviewed journal.

Discussion

To our knowledge, this is the first cluster randomised trial to investigate the effectiveness and safety of the smart phone-based TEAS as adjunctive therapy for hypertension.

This study is a community-based cluster randomised trial. There are several advantages for this design. First, contamination is one of the common biases, but it can be alleviated by the cluster design because only the communities randomly assigned to the TEAS group will have access to the instrument. Furthermore, communities were chosen with

For peer review only - http://bmjopen.bmj.com/site/about/guidelines.xhtml

adequate geographical separation from each other. Second, patients will be recruited from their homes or communities. Unlike hospital-based studies, which only include those who seek medical help, our design will overcome the traditionally low detection of hypertension.

The TEAS in this trial can be received through smart phone at home by the patients themselves. The majority of hypertension patients had to travel by taxi or use public transportation to attend physician visits and prescribe antihypertensive drugs. If the effectiveness of TEAS is verified, this non-pharmaceutical therapy will result in additional savings of cost and burden. The time, expense and lost wages are also identified as barriers for controlling blood pressure. The smart phone-based TEAS at home may contribute to improving treatment adherence. Furthermore, several patients sometimes forget to take medicine, especially when they are busy working or tripping. As we mentioned before, we could use the background of TEAS software to recognise patients with poor compliance real-timely and remind them to complete treatment.

For peer review only

The mechanism of acupuncture to reduce blood pressure is still unclear. Plentz R and colleagues found that TEAS could decrease sympathetic nervous system activity and increase parasympathetic nervous system activity,[31] which may contribute to reducing blood pressure. Moreover, TEAS was shown to have a vasodilator effect.[32] Our animal experiments also indicated that mitogen-activated protein kinases and β-adrenergic receptors were involved in the mechanism of acupuncture's amelioration of

For peer review only - http://bmjopen.bmj.com/site/about/guidelines.xhtml

hypertension.[33 34]

Our study has several limitations. First, blinding for patients was not feasible; however, several steps will be implemented to minimise potential biases. Outcome assessors will be masked and an electronic data capture system with tablet has been developed to separate streams of data collection processes. Moreover, the blood pressure recordings were from automated devices to minimise biases for the key outcome. Second, surrogate outcomes are used in this study. There is good evidence to support improved clinical outcomes with the reductions in blood pressure shown in other trials.[35 36] To show a benefit in clinical events, a much larger and longer trial would be required. Third, patients in the usual care group will be screened for cardiovascular disease risk at baseline, have their blood pressure assessed, and be provided with information about hypertension education. After being aware of their elevated blood pressure, some patients in the usual care group might modify their behavior and receive more treatment than clinical practice. This modification might reduce the real differences between the groups, which means that we may underestimate the effectiveness of TEAS.

This trial may provide high-quality evidence for the effectiveness of the smart phone-based TEAS as adjunctive therapy for hypertension. If the study hypothesis is confirmed, smart phone-based TEAS may help to attain the United Nations General Assembly Action Plan for a one-third reduction in premature mortality from cardiovascular disease.

For peer review only - http://bmjopen.bmj.com/site/about/guidelines.xhtml

Trial status

Recruitment was started at 18th November 2020 and is expected to close in June 2022.

For peer review only

For peer review only - http://bmjopen.bmj.com/site/about/guidelines.xhtml

Acknowledgements

Appreciation to every participant in the trial and every personnel in recruitment sites for their contributions.

Contributors

Liu CZ conceived of the study. Liu CZ, Tu JF, and Wang LQ initiated the study design. Yan SY drew up the statistical plan. Kang SB, Yan CQ, Su XT, Shi GX, Mi BH, Lin Y, Wang Y, Li HW, Yang JW, Wang XZ, and Wang X helped with its implementation. Tu JF and Kang SB drafted the manuscript and all the authors critically revised the manuscript. Liu CZ sought funding. Liu CZ, Tu JF, and Wang LQ sought ethical approval. All authors contributed to the refinement of the study protocol and approved the final manuscript.

Funding

This work was supported by National Key R&D Program of China (grant number: 2019YFC1712100) and Project for Distinguished Young Scholars of Beijing University of Chinese Medicine (grant number: BUCM-2019-JCRC011). The sponsors have no role in the design of the trial, collection, management, analysis, interpretation of data; writing of the report or the decision to submit the report for publication.

Competing interests

None declared.

For peer review only - http://bmjopen.bmj.com/site/about/guidelines.xhtml

Patient consent for publication

Written informed consent for publication of Figure 2 was obtained from the individual. This is available from the corresponding author on request.

Ethics approval

Ethical approval for this study was obtained from the Ethics Committee of Beijing University of Chinese Medicine (2020BZHYLL0104).

Data Availability Statement

There are no data in this article.

For peer review only

For peer review only - http://bmjopen.bmj.com/site/about/guidelines.xhtml

REFERENCES

1. GBD 2016 Risk Factors Collaborators. Global, regional, and national comparative risk assessment of 84 behavioural, environmental and occupational, and metabolic risks or clusters of risks, 1990-2016: a systematic analysis for the Global Burden of Disease Study 2016. *Lancet* 2017;390:1345-422.

2. World Health Organize. Hypertension [EB/OL]. https://www.who.int/health-topics/hypertension/, 2021-9-16.

3. Whelton PK, Carey RM, Aronow WS, *et al.* 2017 ACC/AHA/AAPA/ABC/ACPM/AGS/APhA/ASH/ASPC/NMA/PCNA Guideline for the Prevention, Detection, Evaluation, and Management of High Blood Pressure in Adults: A Report of the American College of Cardiology/American Heart Association Task Force on Clinical Practice Guidelines. *Circulation* 2018;138:e484-484e594.

4. Garies S, Hao S, McBrien K, *et al.* Prevalence of Hypertension, Treatment, and Blood Pressure Targets in Canada Associated With the 2017 American College of Cardiology and American Heart Association Blood Pressure Guidelines. *JAMA Netw Open* 2019;2:e190406.

5. Wang JG, Liu L. Global Impact of 2017 American College of Cardiology/American Heart Association Hypertension Guidelines: A Perspective From China. *Circulation* 2018;137:546-8.

6. Venkateshmurthy NS, Geldsetzer P, Jaacks LM, *et al.* Implications of the New American College of Cardiology Guidelines for Hypertension Prevalence in India.

For peer review only - http://bmjopen.bmj.com/site/about/guidelines.xhtml

JAMA Intern Med 2018;178:1416-8.

7. Lim SS, Vos T, Flaxman AD*, et al.* A comparative risk assessment of burden of disease and injury attributable to 67 risk factors and risk factor clusters in 21 regions, 1990-2010: a systematic analysis for the Global Burden of Disease Study 2010. *Lancet* 2012;380:2224-60.
8. Writing Group Members, Mozaffarian D, Benjamin EJ*, et al.* Heart Disease and Stroke Statistics-2016 Update: A Report From the American Heart Association. *Circulation* 2016;133:e38-360.
9. Williams B, Mancia G, Spiering W*, et al.* 2018 ESC/ESH Guidelines for the management of arterial hypertension. *Eur Heart J* 2018;39:3021-104.
10. Guidelines for the prevention and treatment of hypertension in China (Revised edition in 2018). Chinese Journal of Cardiovascular Medicine [in Chinese] 2019;24:24-56.
11. Zhao L, Li D, Zheng H*, et al.* Acupuncture as Adjunctive Therapy for Chronic Stable Angina: A Randomized Clinical Trial. *JAMA Intern Med* 2019.
12. Wang Q, Liang D, Wang F*, et al.* Efficacy of electroacupuncture pretreatment for myocardial injury in patients undergoing percutaneous coronary intervention: A randomized clinical trial with a 2-year follow-up. *Int J Cardiol* 2015;194:28-35.
13. Flachskampf FA, Gallasch J, Gefeller O*, et al.* Randomized trial of acupuncture to lower blood pressure. *Circulation* 2007;115:3121-9.
14. Zheng H, Li J, Li Y*, et al.* Acupuncture for patients with mild hypertension: A randomized controlled trial. *J Clin Hypertens (Greenwich)* 2019;21:412-20.

For peer review only - http://bmjopen.bmj.com/site/about/guidelines.xhtml

15. Yang J, Chen J, Yang M, *et al.* Acupuncture for hypertension. *Cochrane Database Syst Rev* 2018;11:CD008821.

16. Jacobsson F, Himmelmann A, Bergbrant A, *et al.* The effect of transcutaneous electric nerve stimulation in patients with therapy-resistant hypertension. *J Hum Hypertens* 2000;14:795-8.

17. Williams T, Mueller K, Cornwall MW. Effect of acupuncture-point stimulation on diastolic blood pressure in hypertensive subjects: a preliminary study. *Phys Ther* 1991;71:523-9.

18. Kaada B, Flatheim E, Woie L. Low-frequency transcutaneous nerve stimulation in mild/moderate hypertension. *Clin Physiol* 1991;11:161-8.

19. Tu JF, Wang LQ, Liu JH, *et al.* Home-based transcutaneous electrical acupoint stimulation for hypertension: a randomized controlled pilot trial. *Hypertens Res* 2021;44:1300-6.

20. Chan AW, Tetzlaff JM, Altman DG, *et al.* SPIRIT 2013 statement: defining standard protocol items for clinical trials. *Ann Intern Med* 2013;158:200-7.

21. Wang Y, Shi GX, Tian ZX, *et al.* Transcutaneous electrical acupoint stimulation for high-normal blood pressure: study protocol for a randomized controlled pilot trial. *Trials* 2021;22:140.

22. Silverdal J, Mourtzinis G, Stener-Victorin E, *et al.* Antihypertensive effect of low-frequency transcutaneous electrical nerve stimulation (TENS) in comparison with drug treatment. *Blood Press* 2012;21:306-10.

23. Climie RE, Schultz MG, Nikolic SB, *et al.* Validity and reliability of central blood

For peer review only - http://bmjopen.bmj.com/site/about/guidelines.xhtml

pressure estimated by upper arm oscillometric cuff pressure. *Am J Hypertens* 2012;25:414-20.

24. Shimamoto K, Ando K, Fujita T*, et al.* The Japanese Society of Hypertension Guidelines for the Management of Hypertension (JSH 2014). *Hypertens Res* 2014;37:253-390.

25. Wan E, Yu E, Chin WY*, et al.* Evaluation of the responsiveness of Short Form-12 Health Survey version 2 (SF-12v2) in Chinese patients with hypertension in primary care. *Qual Life Res* 2019;28:2851-7.

26. Hallal PC, Victora CG. Reliability and validity of the International Physical Activity Questionnaire (IPAQ). *Med Sci Sports Exerc* 2004;36:556.

27. Yan J, You LM, Yang Q*, et al.* Translation and validation of a Chinese version of the 8-item Morisky medication adherence scale in myocardial infarction patients. *J Eval Clin Pract* 2014;20:311-7.

28. Food and Drug Administration. Principles for clinical evaluation of new antihypertensive drugs [EB/OL]. https://www.fda.gov/media/71363/download, 2021-9-16.

29. Killip S, Mahfoud Z, Pearce K. What is an intracluster correlation coefficient? Crucial concepts for primary care researchers. *Ann Fam Med* 2004;2:204-8.

30. Li P, Stuart EA, Allison DB. Multiple Imputation: A Flexible Tool for Handling Missing Data. *JAMA* 2015;314:1966-7.

31. do Amaral Sartori S, Stein C, Coronel CC*, et al.* Effects of Transcutaneous Electrical Nerve Stimulation in Autonomic Nervous System of Hypertensive

For peer review only

For peer review only - http://bmjopen.bmj.com/site/about/guidelines.xhtml

Patients: A Randomized Controlled Trial. *Curr Hypertens Rev* 2018;14:66-71.

32. Mannheimer C, Emanuelsson H, Waagstein F. The effect of transcutaneous electrical nerve stimulation (TENS) on catecholamine metabolism during pacing-induced angina pectoris and the influence of naloxone. *Pain* 1990;41:27-34.

33. Wang XR, Yang JW, Ji CS*, et al.* Inhibition of NADPH Oxidase-Dependent Oxidative Stress in the Rostral Ventrolateral Medulla Mediates the Antihypertensive Effects of Acupuncture in Spontaneously Hypertensive Rats. *Hypertension* 2018;71:356-65.

34. Yang JW, Ye Y, Wang XR*, et al.* Acupuncture Attenuates Renal Sympathetic Activity and Blood Pressure via Beta-Adrenergic Receptors in Spontaneously Hypertensive Rats. *Neural Plast* 2017;2017:8696402.

35. Roshandel G, Khoshnia M, Poustchi H*, et al.* Effectiveness of polypill for primary and secondary prevention of cardiovascular diseases (PolyIran): a pragmatic, cluster-randomised trial. *Lancet* 2019;394:672-83.

36. Yusuf S, Lonn E, Pais P*, et al.* Blood-Pressure and Cholesterol Lowering in Persons without Cardiovascular Disease. *N Engl J Med* 2016;374:2032-43.

For peer review only

For peer review only - http://bmjopen.bmj.com/site/about/guidelines.xhtml

Table 1 Locations of acupoints in the TEAS group

Acupoints	Locations
Taichong (LR3)	On the dorsum of the foot, between the first and second metatarsal bones, in the depression distal to the junction of the bases of the two bones, over the dorsalis pedis artery.
Zusanli (ST36)	On the anterior aspect of the leg, on the line connecting ST35 with ST41, 3 cun inferior to ST35.
Hegu (LI4)	On the dorsum of the hand, radial to the midpoint of the second metacarpal bone.
Quchi (LI11)	On the lateral aspect of the elbow, at the midpoint of the line connecting LU5 with the lateral epicondyle of the humerus.

[a] 1 cun (≈20 mm) is defined as the width of the interphalangeal joint of patient's thumb.

For peer review only

Figure legends

Figure 1 Flow diagram

TEAS, transcutaneous electrical acupoint stimulation.

Figure 2 The instrument and the locations of acupoints

Figure 3 Schedule of enrollment, intervention and assessments

TEAS, transcutaneous electrical acupoint stimulation.

For peer review only

For peer review only - http://bmjopen.bmj.com/site/about/guidelines.xhtml

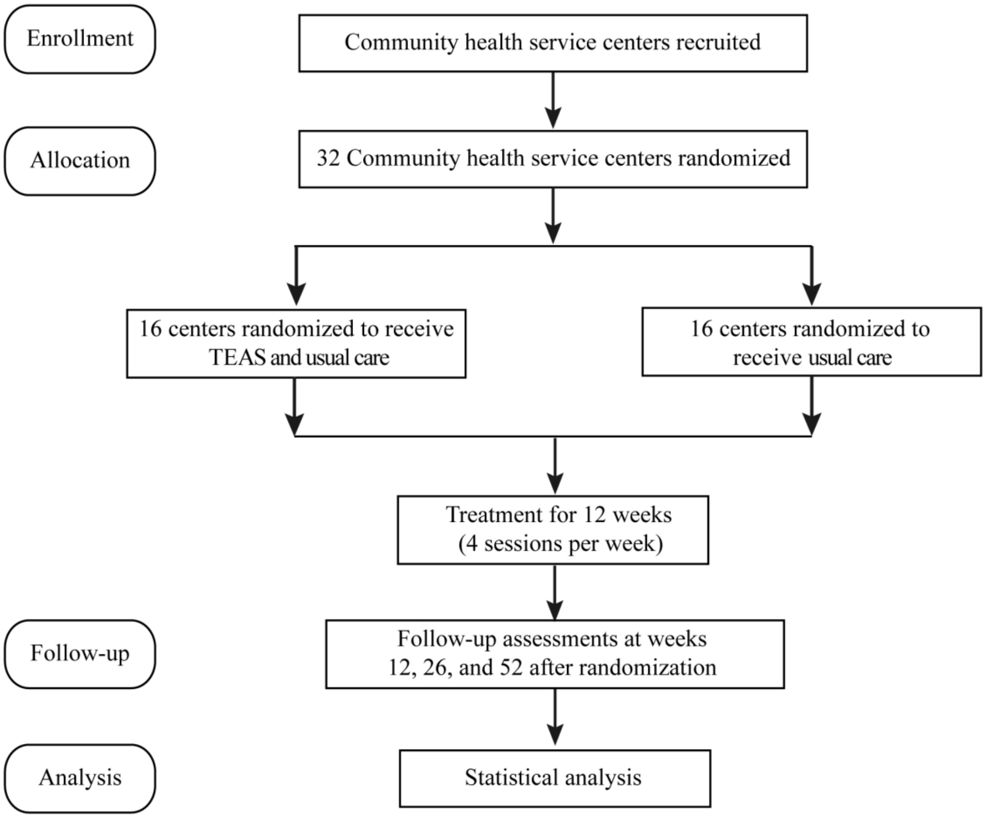

Figure 1 Flow diagram
TEAS, transcutaneous electrical acupoint stimulation.

177x147mm (300 x 300 DPI)

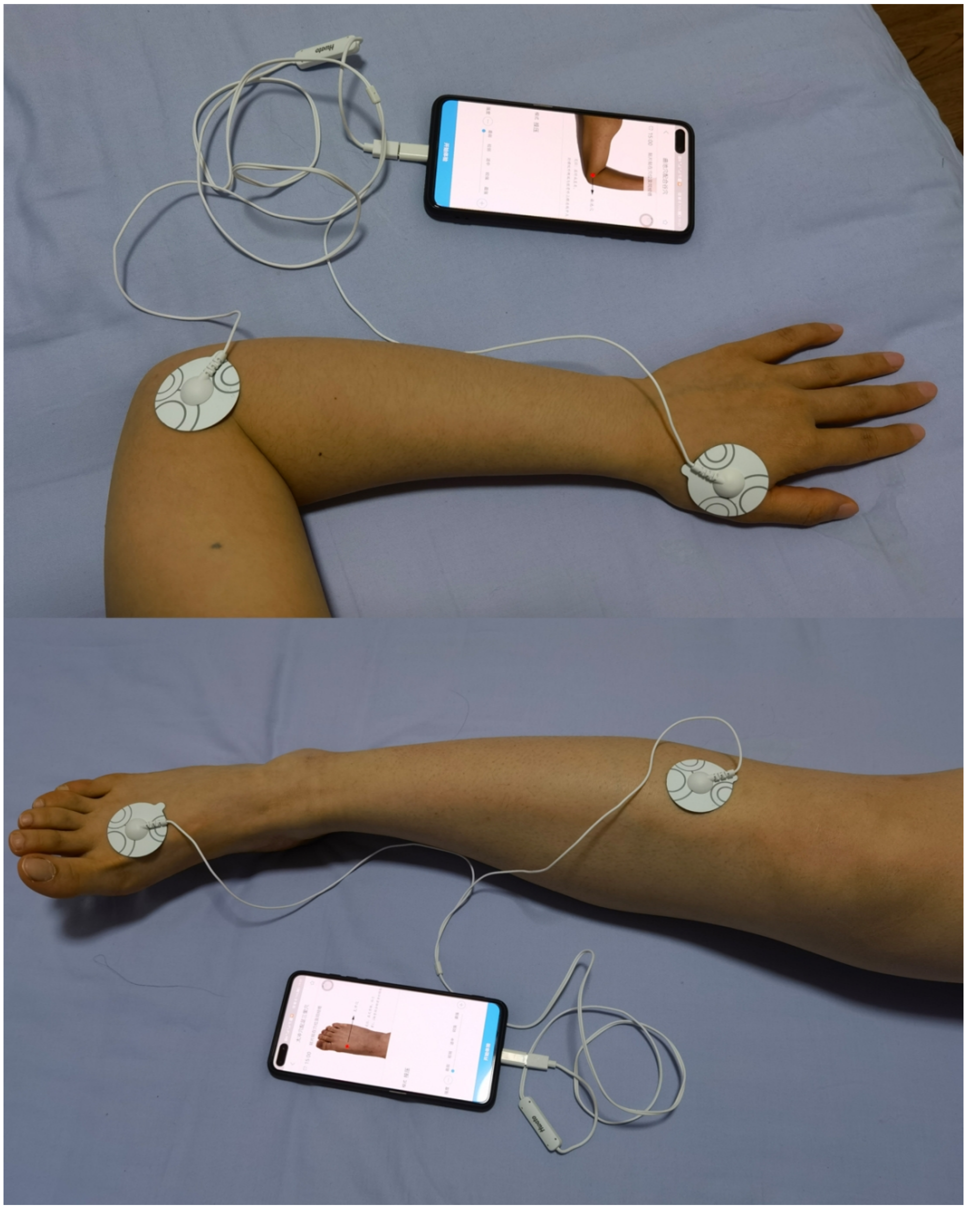

Figure 2 The instrument and the locations of acupoints

101x127mm (300 x 300 DPI)

	STUDY PERIOD				
	Enrolment	Allocation	Post-allocation		Closeout
TIME POINT	Week -1	Week 0	Week 12	Week 26	Week 52
ENROLLMENT:					
[Eligibility screen]	×				
[Informed consent]	×				
[Randomization]		×			
INTERVENTION:					
[TEAS+usual care]		◆———	———◆		
[usual care]		◆———	———◆		
ASSESSMENTS:					
[systolic blood pressure]		×	×	×	×
[diastolic blood pressure]		×	×	×	×
[blood pressure control rate]		×	×	×	×
[proportion of patients taking antihypertensive drugs]		×	×	×	×
[number of antihypertensive drugs]		×	×	×	×
[12-Item Short-Form]		×	×	×	×
[body mass index]		×	×	×	×
[waist circumference]		×	×	×	×
[physical activity]		×	×	×	×
[medication adherence]		×	×	×	×
[blinding test]			×		
[serious adverse events]			×	×	×
[clinical events]			×	×	×

Figure 3 Schedule of enrollment, intervention and assessments
TEAS, transcutaneous electrical acupoint stimulation.

188x156mm (300 x 300 DPI)

eTable 1 The list of Community Health Service Centers

Number	Institution	District
1	Jianguomen Community Health Service Center	Dongcheng District
2	Hepingli Community Health Service Center	Dongcheng District
3	Shichahai Community Health Service Center	Xicheng District
4	Yuetan Community Health Service Center	Xicheng District
5	Sunhe Community Health Service Center	Chaoyang District
6	Cuigezhuang Second Community Health Service Center	Chaoyang District
7	Nanyuan Community Health Service Center	Fengtai District
8	Dahongmen Community Health Service Center	Fengtai District
9	Jindingjie Community Health Service Center	Shijingshan District
10	Guangning Street Community Health Service Center	Shijingshan District
11	Qinglongqiao Community Health Service Center	Haidian District
12	Malanwa Community Health Service Station	Haidian District
13	Tianzhu Community Health Service Center	Shunyi District
14	Zhaoquanying Community Health Service Center	Shunyi District
15	Yongshun Community Health Service Center	Tongzhou District
16	Taihu Community Health Service Center	Tongzhou District
17	Xihongmen Community Health Service Center	Daxing District
18	Changziying Community Health Service Center	Daxing District
19	Liangxiang Community Health Service Station	Fangshan District
20	Changyang Community Health Service Center	Fangshan District

For peer review only

21	Junzhuang Community Health Service Center	Mentougou District
22	Zaitang Community Health Service Center	Mentougou District
23	Shahe Community Health Service Center	Changping District
24	Huilongguan Community Health Service Center	Changping District
25	Huangsongyu Community Health Service Center	Pinggu District
26	Yukou Community Health Service Center	Pinggu District
27	Henanzhai Community Health Service Center	Miyun District
28	Mujianyu Community Health Service Center	Miyun District
29	Yangsong Community Health Service Center	Huairou District
30	Miaocheng Community Health Service Center	Huairou District
31	Zhangshanying Community Health Service Center	Yanqing District
32	Yongning Community Health Service Center	Yanqing District

For peer review only

STANDARD PROTOCOL ITEMS: RECOMMENDATIONS FOR INTERVENTIONAL TRIALS

SPIRIT 2013 Checklist: Recommended items to address in a clinical trial protocol and related documents*

Section/item	Item No	Description	Page
**Administrative information**			
Title	1	Descriptive title identifying the study design, population, interventions, and, if applicable, trial acronym	1
Trial registration	2a	Trial identifier and registry name. If not yet registered, name of intended registry	4
	2b	All items from the World Health Organization Trial Registration Data Set	7-13
Protocol version	3	Date and version identifier	7,16
Funding	4	Sources and types of financial, material, and other support	20
Roles and responsibilities	5a	Names, affiliations, and roles of protocol contributors	1,20
	5b	Name and contact information for the trial sponsor	20
	5c	Role of study sponsor and funders, if any, in study design; collection, management, analysis, and interpretation of data; writing of the report; and the decision to submit the report for publication, including whether they will have ultimate authority over any of these activities	20
	5d	Composition, roles, and responsibilities of the coordinating centre, steering committee, endpoint adjudication committee, data management team, and other individuals or groups overseeing the trial, if applicable (see Item 21a for data monitoring committee)	15

For peer review only

SPIRIT 2013 Checklist: Recommended items to address in a clinical trial protocol and related documents*(continued)

Section/item	Item No	Description	Page
**Introduction**			
Background and rationale	6a	Description of research question and justification for undertaking the trial, including summary of relevant studies (published and unpublished) examining benefits and harms for each intervention	6-7
	6b	Explanation for choice of comparators	6-7
Objectives	7	Specific objectives or hypotheses	7
Trial design	8	Description of trial design including type of trial (eg, parallel group, crossover, factorial, single group), allocation ratio, and framework (eg, superiority, equivalence, noninferiority, exploratory)	7
**Methods: Participants, interventions, and outcomes**			
Study setting	9	Description of study settings (eg, community clinic, academic hospital) and list of countries where data will be collected. Reference to where list of study sites can be obtained	7,Supplement 1
Eligibility criteria	10	Inclusion and exclusion criteria for participants. If applicable, eligibility criteria for study centres and individuals who will perform the interventions (eg, surgeons, psychotherapists)	8-9
Interventions	11a	Interventions for each group with sufficient detail to allow replication, including how and when they will be administered	10-11
	11b	Criteria for discontinuing or modifying allocated interventions for a given trial participant (eg, drug dose change in response to harms, participant request, or improving/worsening disease)	10

For peer review only - http://bmjopen.bmj.com/site/about/guidelines.xhtml

SPIRIT 2013 Checklist: Recommended items to address in a clinical trial protocol and related documents*(continued)

Section/item	Item No	Description	Page
	11c	Strategies to improve adherence to intervention protocols, and any procedures for monitoring adherence (eg, drug tablet return, laboratory tests)	11
	11d	Relevant concomitant care and interventions that are permitted or prohibited during the trial	10
Outcomes	12	Primary, secondary, and other outcomes, including the specific measurement variable (eg, systolic blood pressure), analysis metric (eg, change from baseline, final value, time to event), method of aggregation (eg, median, proportion), and time point for each outcome. Explanation of the clinical relevance of chosen efficacy and harm outcomes is strongly recommended	12-13
Participant timeline	13	Time schedule of enrolment, interventions (including any run-ins and washouts), assessments, and visits for participants. A schematic diagram is highly recommended (see Figure)	7, Figure 1 and 3
Sample size	14	Estimated number of participants needed to achieve study objectives and how it was determined, including clinical and statistical assumptions supporting any sample size calculations	13
Recruitment	15	Strategies for achieving adequate participant enrolment to reach target sample size	7-8
**Methods: Assignment of interventions (for controlled trials)**			
Allocation:			
Sequence generation	16a	Method of generating the allocation sequence (eg, computer-generated random numbers), and list of any factors for stratification. To reduce predictability of a random sequence, details of any planned restriction (eg, blocking) should be provided in a separate document that is unavailable to those who enrol participants or assign interventions	9

For peer review only - http://bmjopen.bmj.com/site/about/guidelines.xhtml

SPIRIT 2013 Checklist: Recommended items to address in a clinical trial protocol and related documents*(continued)

Section/item	Item No	Description	Page
Allocation concealment mechanism	16b	Mechanism of implementing the allocation sequence (eg, central telephone; sequentially numbered, opaque, sealed envelopes), describing any steps to conceal the sequence until interventions are assigned	9
Implementation	16c	Who will generate the allocation sequence, who will enrol participants, and who will assign participants to interventions	9
Blinding (masking)	17a	Who will be blinded after assignment to interventions (eg, trial participants, care providers, outcome assessors, data analysts), and how	9
	17b	If blinded, circumstances under which unblinding is permissible, and procedure for revealing a participant's allocated intervention during the trial	9
**Methods: Data collection, management, and analysis**			
Data collection methods	18a	Plans for assessment and collection of outcome, baseline, and other trial data, including any related processes to promote data quality (eg, duplicate measurements, training of assessors) and a description of study instruments (eg, questionnaires, laboratory tests) along with their reliability and validity, if known. Reference to where data collection forms can be found, if not in the protocol	12-13
	18b	Plans to promote participant retention and complete follow-up, including list of any outcome data to be collected for participants who discontinue or deviate from intervention protocols	12
Data management	19	Plans for data entry, coding, security, and storage, including any related processes to promote data quality (eg, double data entry; range checks for data values). Reference to where details of data management procedures can be found, if not in the protocol	15
Statistical methods	20a	Statistical methods for analysing primary and secondary outcomes. Reference to where other details of the statistical analysis plan can be found, if not in the protocol	14-15

SPIRIT 2013 Checklist: Recommended items to address in a clinical trial protocol and related documents*(continued)

Section/item	Item No	Description	Page
	20b	Methods for any additional analyses (eg, subgroup and adjusted analyses)	14-15
	20c	Definition of analysis population relating to protocol non-adherence (eg, as randomised analysis), and any statistical methods to handle missing data (eg, multiple imputation)	14-15
**Methods: Monitoring**			
Data monitoring	21a	Composition of data monitoring committee (DMC); summary of its role and reporting structure; statement of whether it is independent from the sponsor and competing interests; and reference to where further details about its charter can be found, if not in the protocol. Alternatively, an explanation of why a DMC is not needed	15
	21b	Description of any interim analyses and stopping guidelines, including who will have access to these interim results and make the final decision to terminate the trial	Not applicable
Harms	22	Plans for collecting, assessing, reporting, and managing solicited and spontaneously reported adverse events and other unintended effects of trial interventions or trial conduct	13
Auditing	23	Frequency and procedures for auditing trial conduct, if any, and whether the process will be independent from investigators and the sponsor	15
**Ethics and dissemination**			
Research ethics approval	24	Plans for seeking research ethics committee/institutional review board (REC/IRB) approval	16
Protocol amendments	25	Plans for communicating important protocol modifications (eg, changes to eligibility criteria, outcomes, analyses) to relevant parties (eg, investigators, REC/IRBs, trial participants, trial registries, journals, regulators)	Not applicable

For peer review only - http://bmjopen.bmj.com/site/about/guidelines.xhtml

SPIRIT 2013 Checklist: Recommended items to address in a clinical trial protocol and related documents*(continued)

Section/item	Item No	Description	Page
Consent or assent	26a	Who will obtain informed consent or assent from potential trial participants or authorised surrogates, and how (see Item 32)	16
	26b	Additional consent provisions for collection and use of participant data and biological specimens in ancillary studies, if applicable	Not applicable
Confidentiality	27	How personal information about potential and enrolled participants will be collected, shared, and maintained in order to protect confidentiality before, during, and after the trial	15
Declaration of interests	28	Financial and other competing interests for principal investigators for the overall trial and each study site	20
Access to data	29	Statement of who will have access to the final trial dataset, and disclosure of contractual agreements that limit such access for investigators	21
Ancillary and post-trial care	30	Provisions, if any, for ancillary and post-trial care, and for compensation to those who suffer harm from trial participation	Not applicable
Dissemination policy	31a	Plans for investigators and sponsor to communicate trial results to participants, healthcare professionals, the public, and other relevant groups (eg, via publication, reporting in results databases, or other data sharing arrangements), including any publication restrictions	16
	31b	Authorship eligibility guidelines and any intended use of professional writers	20
	31c	Plans, if any, for granting public access to the full protocol, participant-level dataset, and statistical code	Not applicable

For peer review only - http://bmjopen.bmj.com/site/about/guidelines.xhtml

SPIRIT 2013 Checklist: Recommended items to address in a clinical trial protocol and related documents*(continued)

Section/item	Item No	Description	Page
**Appendices**			
Informed consent materials	32	Model consent form and other related documentation given to participants and authorised surrogates	Consent Form
Biological specimens	33	Plans for collection, laboratory evaluation, and storage of biological specimens for genetic or molecular analysis in the current trial and for future use in ancillary studies, if applicable	Not applicable

*It is strongly recommended that this checklist be read in conjunction with the SPIRIT 2013 Explanation & Elaboration for important clarification on the items. Amendments to the protocol should be tracked and dated. The SPIRIT checklist is copyrighted by the SPIRIT Group under the Creative Commons "Attribution-NonCommercial-NoDerivs 3.0 Unported" license.

For peer review only
